# Parenting practices and child irritability across diverse racial–ethnic backgrounds: A temporal network analysis

**DOI:** 10.1017/S095457942610145X

**Published:** 2026-05-05

**Authors:** Yi Voon Lim, Nellia Bellaert, Nabihah Ahsan, Kalina Michalska, Wan-Ling Tseng

**Affiliations:** 1 Sewanee The University of the South, USA; 2 Yale University, USA; 3 Service de Psychologie Cognitive et Neuropsychologie, Université de Mons, Belgium; 4 University of California Riverside, USA; 5 Yale Child Study Center, USA

**Keywords:** Cross-cultural, irritability, longitudinal, parenting behaviors, parenting stress

## Abstract

Irritability is a core symptom and diagnostic criterion in several childhood psychiatric disorders. Research has documented bidirectional associations between child irritability and parenting practices; however, cultural variations in these associations remain underexplored.

Using three-wave longitudinal data (*N* = 2,408) from the Future of Families and Child Wellbeing Study (FFCWS) in the United States, this study examined associations between child irritability, parenting behaviors (psychological aggression, physical assault, neglect, and non-violent discipline) and parenting stress across three racial–ethnic groups: non-Latine Black (*n* = 1,167; 605 males), non-Latine White (*n* = 614; 314 males), and Latine (*n* = 627; 316 males) using cross-sectional and temporal network analyses.

Parenting behaviors and stress were associated with child irritability concurrently and longitudinally across groups. Results showed bidirectional effects between parenting behaviors/stress and child irritability across ages 3, 5, and 9, with more similarities than differences between groups. Physical assault and lower use of non-violent discipline predicted higher future child irritability (partial correlations = 0.03–0.18 for physical assault and 0.04–0.07 for non-violent discipline) across racial–ethnic groups.

These findings suggest parenting interventions may be scalable across cultural contexts to promote positive child outcomes and well-being, though future work should elucidate culturally specific factors that inform tailored practices.

## Introduction

Irritability is a common symptom and mood experienced by individuals across different developmental periods. It refers to a state of heightened sensitivity or a tendency to react with annoyance, anger, or frustration to minor provocations (Leibenluft et al., 2024; Stringaris et al., 2018). In early childhood, irritability may manifest as aggression, temper loss, and non-compliance (Wakschlag et al., 2009) and is a robust transdiagnostic marker of later emotional and behavioral syndromes (Wakschlag et al., 2019). Indeed, irritability is the most common reason families seek mental health care and treatment for youth (Evans et al., 2022). Thus, identifying and addressing irritability in early childhood is important to promote positive outcomes later in life (Wakschlag et al., 2022).

Parent-child relationships and interactions constitute an important, immediate environmental context for early child development. Sameroff’s transactional model of development (Sameroff, 2009) posits that development reflects continuous dynamic exchanges between the child and their environment over time, with influences that are interdependent and bidirectional. Consistent with this framework, children with severe irritability are more likely to experience negative parenting behaviors (Wiggins et al., 2014), and the association between parenting and child irritability appears bidirectional (Lee et al., 2023; Lengua & Kovacs, 2005). That is, while negative parenting behaviors may contribute to child irritability (Ravi et al., 2022), an irritable child may also elicit more parental distress and harsher responses (Lee et al., 2023; Lengua & Kovacs, 2005). This is well-illustrated in Patterson’s parent-child coercive cycle (Patterson, 2002) where a child’s misbehavior is met with harsh parenting, further evoking child misbehavior, and the negative cycle continues. Investigating how specific parenting behaviors influence irritability, and vice versa, over time may inform targeted prevention and intervention efforts to break the negative parent-child coercive cycle and support healthy child development.

Past research linking parenting and child irritability has focused on inconsistent and harsh parenting, including psychological and physical aggression (Derella et al., 2020; Valencia et al., 2021; Wiggins et al., 2014). Psychological aggression, involving verbal threats, insults, and emotional rejection, can undermine a child’s emotional well-being (Hibbard et al., 2012) and predicts increases in child irritability (Derella et al., 2020). Physical aggression, which involves the application of physical force or violence towards a child (e.g., hitting, spanking), can also increase child irritability. In the Pittsburgh Girls Study, corporal punishment predicted increases in child irritability over time (Derella et al., 2020). Research has also documented a link between corporal punishment and anger/aggression, a common correlate of irritability (see Gershoff, 2002 for a review). Neglect, characterized by low levels of involvement, emotional support, and supervision, may impede emotion regulation and exacerbate irritability (Ravi et al., 2022). In contrast, non-violent discipline strategies, such as time-outs, removal of privileges, and explaining appropriate behavior (Quail & Ward, 2023), are endorsed by the American Academy of Pediatrics as constructive alternatives that foster children’s cognitive, socioemotional, and executive functioning skills (Sege et al., 2018).

Coll’s integrative model of child development (Coll et al., 1996) posits that children’s developmental competencies emerge from the interplay between adaptive culture (e.g., traditions, cultural legacies, migration, and acculturation) and family-level factors (e.g., family values, beliefs, structures, and roles), which provide pathways to children’s developmental competencies. Cultural norms, values, beliefs, traditions, and socioeconomic conditions shape how parents appraise irritability and approach caregiving and discipline (Breiner et al., 2024). Consistent with Bornstein’s distinction between “form” and “function” of parenting (Bornstein, 2012), parents from different cultural groups may engage in the same parenting behavior (form) for different purposes, or conversely different behaviors that serve similar regulatory goals (Bornstein, 2012). For example, European-American and Puerto Rican mothers differ in how they value individual autonomy versus interdependence (Bornstein, 2012), influencing their parenting styles. European-American mothers more often guide their children’s behavior through suggestions, whereas Puerto Rican mothers more frequently use direct commands, physical positioning, and restraints (Bornstein, 2012). Differences are also reported in disciplinary practices: non-Latine Black parents are about twice as likely as non-Latine White parents to report spanking, whereas Latine parents report lower use of non-violent discipline than non-Latine White parents (Regalado et al., 2004). Given that physical aggression is linked to later irritability (Derella et al., 2020), and non-violent discipline supports socioemotional development (Sege et al., 2018), understanding how culturally shaped parenting behaviors relate to irritability is essential. Generational and social trends within cultural contexts shape parenting behaviors and practices, with downstream implications for children’s psychological development (Kang et al., 2022).

Consistent with Coll and colleagues (1996), it is important to consider the normative experiences of minoritized groups when examining associations between parenting behaviors and children’s socioemotional difficulties. Yet, cross-cultural evidence on irritability is limited. To our knowledge, the only relevant study, a longitudinal cross-national study spanning twelve ethnic-cultural groups, found more similarities than differences in how parental irritability-related behaviors and harsh discipline correlated with adolescent adjustment (Di Giunta et al., 2020). Specifically, Di Giunta et al. (2020) noted that maternal and paternal harsh parenting at ages 13 and 14 was correlated with adolescent irritability at age 14, and this was invariantly observed across the twelve examined ethnic-cultural groups. This finding complicates expectations derived from Coll’s integrative model (1996); although cultural context clearly matters, some associations between harsh parenting and youth irritability may be robust across cultures. More recent cross-cultural work shows that, despite no substantial variation in irritability frequency, associations between irritability and outcomes such as life satisfaction and bullying can vary by country, underscoring the importance of identifying culture-specific moderators (Silver et al., 2025). In the present study, we therefore examined whether associations between specific parenting behaviors and child irritability differ across racial–ethnic groups within the United States (US).

The extent to which parenting behaviors are employed varies with other contextual factors such as external stress (e.g., unemployment, stressful life events, racial discrimination) (Breiner et al., 2024). External stressors may undermine optimal parenting and contribute to parenting stress – defined as a parent’s available coping resources being insufficient to meet parenting demands (Nomaguchi & House, 2013). In addition to maladaptive parenting behaviors, high levels of parenting stress are associated with poor child outcomes, such as behavior problems (Hofferth et al., 1997). Research has indicated cultural and racial-ethnic differences in parenting stress (Chao & Kanatsu, 2008). For example, in the US, non-Latine Black and Latine mothers report higher levels of parenting stress than non-Latine White mothers, often due to systemic structural disadvantages like lower family income, limited English proficiency, and experiences of racial-ethnic prejudice as well as authoritarian parenting values that may heighten frustration and conflict with children (Nomaguchi & House, 2013). In addition to its direct relationship with child behavior problems (Jackson & Choi, 2018), elevated external or parenting stress in minoritized racial-ethnic groups may lead to more punitive parental reactions, precipitate stricter rule-enforcement practices intended to keep children safe (Cooper et al., 2020), and potentially confer differential risks of psychopathology symptoms in children (Chan et al., 2018). Nonetheless, few studies have examined how parenting stress directly or indirectly (via negative parenting) relates to child irritability or whether any observed associations vary across cultural contexts, which likely reflects, in part, structural inequities as well as cultural norms.

Parenting is shaped in part by parents’ cultural values, which vary across families from racial-ethnic minority and majority backgrounds (Suizzo, 2007). Cultural normativeness, the extent to which a behavior is accepted and widely practiced in a given context, may moderate the associations between harsh parenting behaviors and child outcomes (Lansford, 2021). Prior research shows cross-cultural variability in how teachers, parents, and youth rate children’s emotional and behavioral problems (Rescorla et al., 2012; Sourander et al., 2024). Although many symptom measures demonstrate configural and metric invariance across cultural groups indicating consistent structure and interpretation, normative cultural perceptions of irritability may still differ meaningfully across contexts (Rescorla et al., 2012; Sourander et al., 2024). Such differences may reflect cultural specificity in how emotional and behavioral problems are expressed and perceived, such that racial–ethnic groups may respond differently to the same child behaviors. In line with Coll’s integrative model (1996), variations in parental responses to identical behaviors can be understood as emerging from the joint influence of adaptive culture and family on developmental outcomes.

Whereas most previous studies have examined broad behavioral problems and symptom dimensions, the present study focuses specifically on irritability, offering a novel, more targeted lens on how it may vary across diverse environments. We investigate associations among parenting behaviors, parenting stress, and child irritability across three racial-ethnic groups (Non-Latine Black, non-Latine White, and Latine) using a large publicly available dataset described below. We evaluate how the strength and pattern of the association might vary across groups (e.g., certain parenting behaviors may be linked to child irritability in some groups but not in other groups). We recognize that race/ethnicity are, at best, imperfect, indirect proxies for cultural values and beliefs; nevertheless, documenting group differences and similarities can lay the groundwork for future studies that incorporate direct measures of values, beliefs, and culturally-specific risk and promotive factors to clarify how cultural and environmental contexts shape parenting practices and child irritability.

Using three-wave longitudinal data from the Future of Families and Child Wellbeing Study (FFCWS), the current study extends previous studies (including those using FFCWS data; Wiggins et al., 2014; Xu et al., 2018) by testing both concurrent and longitudinal associations between child irritability and parenting behaviors and stress across multiple racial-ethnic groups. In addition, this study also builds upon Di Giunta and colleagues’ (2020) work by (1) differentiating distinct components of harsh parenting; (2) focusing on child, instead of parent, irritability; (3) examining an earlier developmental period (ages 3 to 9 instead of ages 13 to 15); and (4) using an innovative statistical approach, i.e., (network analysis). We apply novel cross-sectional and temporal network analyses (Epskamp, 2020) to characterize the complex interrelations between child irritability and parenting behaviors/stress within and between groups. This approach moves beyond traditional variable-centered, often cross-sectional, models of parenting and emotion regulation. Compared with commonly used longitudinal modeling techniques such as cross-lagged panel models, network analyses better separate within-and between-person effects, reducing ambiguity in inferences about within-person and between-person processes across development (Bellaert et al., 2023). They also allow visualization of nuanced interactions between variables and captures bidirectional relationships, unlike traditional regression and structural equation models, which assume unidirectional effects. Finally, temporal network models support exploratory analyses grounded in group differences to reveal the temporal, dynamic, complex interactions and patterns between all variables in the network – an advantage over models that handle a small set of variables and require clear directional hypotheses. They also more easily incorporate stationarity assumptions to capture consistent temporal effects across timepoints, helping to estimate stable rather than time-specific effects.

We hypothesized that parenting stress, psychological aggression, physical assault, and neglect would be bidirectionally positively associated with child irritability over time, whereas non-violent discipline would be bidirectionally inversely associated with child irritability over time. We further expected differences in the strength and configuration of these associations across racial-ethnic groups, as parenting behaviors, stress, and their associations with child outcomes may vary across groups; at the same time, based on prior work, we anticipated that some associations would be more similar than different across groups (Di Giunta et al., 2020).

## Method

### Participants

Participant data were drawn from the FFCWS, an ongoing stratified and longitudinal study of 4,898 children born in 20 major US cities between 1998–2000, oversampling births to unmarried mothers by a ratio of 3:1 (see Reichman et al., 2001 for full details on sample design). Data were obtained at four timepoints: ages 0 (baseline), 3, 5, and 9. Baseline participation consisted of 4,898 participants with an attrition rate of 22.15% by age 9. Attrition occurred across waves as follows: baseline to age 3, 10.88%; age 3 to age 5, 1.43%; age 5 to age 9, 9.84%. The final analytic sample included data from 2,408 mothers from three racial-ethnic groups: Non-Latine Black (*n* = 1,167; referred to hereafter as Black), Non-Latine White (*n* = 614; referred to hereafter as White), and Latine (*n* = 627) participants.

The FFCWS design consisted of different surveys for mothers and primary childcare givers (PCGs). The mother was considered the primary caregiver if she lived with the child at least half of the time. Overall, mothers provided >92% of PCG reports across all timepoints. Because measures of interest were administered in different respondent surveys, we combined mother and PCG surveys. At age 3, mothers reported parenting stress and PCGs (98.89% mothers, 1.11% other caregivers) reported child irritability and parenting behaviors. At age 5, mothers reported parenting stress and child irritability, and PCGs (97.83% mothers, 2.17% other caregivers) reported parenting behaviors. At age 9, PCGs (92.42% mothers, 7.58% other caregivers) reported parenting stress, parenting behaviors and child irritability. Mothers also reported their race-ethnicity and cultural belonging (i.e., cultural attachment and cultural practice) at age 0.

Participants were included if they had complete irritability data at ≥2 timepoints, completed the two cultural belonging items (both parents), and identified as one of the three majority represented race-ethnicity groups in this study (Black, White, and Latine). Other racial-ethnic groups (e.g., Asian, Native American, mixed-race populations) were not included due to insufficient sample sizes. The analytic sample (*N* = 2,408) did not differ from the excluded sample (*N* =2,490) in child gender or mother age (*p*’s ≥.22); however, they differed in mothers’ relationship status, education, household income, and race (*p*’s < .001; see Table S1). Table 1 summarizes sociodemographic characteristics of the final analytic sample. The three groups did not differ in child gender (*p* ≥.05); however, they differed in mothers’ age, education, relationship status, and household income (*p*’s < .001).

**Table 1.** Socio-demographic characteristics of the final sample

Socio-demographic variable	White participants (*N* = 614)	Black participants (*N* = 1,167)	Latine participants (*N* = 627)	Total sample (*N* = 2,408)
		Mean (SD)		
Mother age at child age 1	27.38 (6.56)	24.44 (5.67)	24.58 (5.65)	25.22 (6.04)
		n (%)		
Child gender				
Male Female	314 (51.14) 300 (48.86)	605 (51.84) 562 (48.16)	316 (50.40) 311 (49.60)	1,235 (51.29) 1,173 (48.71)
Mother relationship status				
Married Cohabitation Visiting Friends Hardly talk, never talk or father unknown	326 (53.09) 204 (33.22) 54 (8.79) 16 (2.61) 14 (2.28)	172 (14.74) 470 (40.27) 430 (36.80) 64 (5.48) 31 (2.66)	168 (26.79) 312 (49.76) 111 (17.70) 17 (2.71) 19 (3.03)	666 (27.65) 986 (40.95) 595 (24.71) 94 (3.90) 64 (2.66)
Mother education				
Less than high school	101 (16.45)	353 (30.25)	291 (46.41)	745 (30.94)
High school	153 (24.92)	430 (36.85)	158 (25.20)	741 (30.77)
Some college or technical/trade school	163 (26.55)	315 (26.99)	149 (23.76)	627 (26.04)
College or graduate/professional school	197 (32.08)	68 (5.83)	27 (4.31)	292 (12.13)
No answer	0 (0)	1 (0.09)	2 (0.32)	3 (0.12)
Mother household income ($)				
<25,000 25,000–50,000 50,000–75,000 75,000–100,000 >100,000	181 (29.48) 169 (27.52) 135 (21.99) 3 (0.49) 126 (20.52)	693 (59.38) 334 (28.62) 103 (8.83) 1 (0.09) 36 (3.08)	363 (57.89) 165 (26.32) 64 (10.21) 1 (0.16) 34 (5.42)	1,237 (54.65) 668 (26.79) 302 (11.05) 5 (0.21) 196 (7.27)

### Measures

#### Irritability

Children’s irritability was measured using three Child Behavior Checklist (CBCL; Achenbach, 1999) items (“tantrums or hot temper,” “sudden changes in mood or feelings,” and “stubborn, sullen or irritable” at all timepoints) completed by PCGs at ages 3 and 9, and mothers at age 5. Age-applicable normed CBCL versions were administered at different timepoints (CBCL/2–3 at age 3, CBCL/4–18 at age 5, and CBCL/6–18 at age 9). Items were rated on a 3-point Likert scale over the past 6 months. Irritability was calculated as the average score of the irritability items, which showed adequate reliability (e.g., internal consistency and test-retest reliability) and validity (e.g., convergent, discriminant; Tseng et al., 2017). McDonald’s omega (Trizano-Hermosilla & Alvarado, 2016) values in the current sample were 0.69, 0.70, and 0.73 at age 3, 5, and 9, respectively. Omega, rather than Cronbach’s alpha, was used in this study because we do not assume tau equivalence in terms of factor loadings (McNeish, 2017).

### Parenting behavior

Parenting behavior was measured using the Parent–Child Conflict Tactics Scale (CTSPC; Straus et al., 1998), a 19-item parent-report measure assessing conflict between a parent and child. The CTSPC has four subscales: non-violent discipline, psychological aggression, physical assault, and neglect. For each item, PCGs responded to the question, “How many times have you done this in the past year?” and rated their behavior on a Likert scale (1 = “once”; 2 = “twice”; 3 =“3–5 times”; 4 = “6–10 times”; 5 = “11–20 times”; 6 = “more than 20 times.” Scores of 7 [“not in the past year, but it happened before”] and 8 [“this has never happened”] were recoded as 0). Non-violent discipline scores were reverse coded so that higher scores reflected fewer non-violent discipline occurrences, aligning them with the other subscales consisting of negative parenting behaviors. The CTSPC scale has acceptable reliability with kappa estimates greater than 0.75 (Straus et al., 1998). Scores for the four parenting subscales were calculated by averaging item scores within each subscale. In the current sample, the McDonald’s omega values for non-violent discipline were 0.75, 0.77, and 0.87 at age 3, 5 and 9, respectively; the omega values for psychological aggression were 0.62, 0.65, and 0.76, respectively; the omega values for physical assault were 0.62, 0.65, and 0.73, respectively; the omega values for neglect were 0.67, 0.65, and 0.72, respectively.

#### Parenting stress

Parenting stress was measured using the parent-report Aggravation in Parenting Scale, which contains items from the Child Development Supplement of the Panel Study of Income Dynamics (Hofferth et al., 1997) assessing the amount of parenting stress associated with changes in employment, income, and other life events. In the FFCWS study, four items were used to specifically assess stress related to parenting at ages 3–9. These items were “Being a parent is harder than I thought it would be,” “I feel trapped by my responsibilities as a parent,” “I find that taking care of my child(ren) is much more work than pleasure,” and “I often feel tired, worn out, or exhausted from raising a family.” Items were assessed using mother-report at ages 3 and 5 and PCG-report at age 9. Responses were rated on a 4-point Likert scale (from 1= strongly agree to 4 = strongly disagree). Scores were reverse-coded (so that higher scores corresponded to higher parenting stress) and averaged, as suggested by the FFCWS scales and concepts documentation for the Aggravation in Parenting scale. McDonald’s omega values in the current sample were 0.68, 0.70, and 0.72 at age 3, 5, and 9, respectively.

#### Cultural belonging

In Coll et al.’s integrative model (1996), adaptive culture, particularly migration and acculturation, influence children’s developmental competencies. To capture this dimension, we included cultural belonging in our cross-sectional networks analysis as an index of mothers’ affiliation with and participation in their cultural group while examining racial-ethnic group differences. Mothers reported their cultural belonging at the baseline of the FFCWS study (age 0). Two questions assessed cultural attachment (i.e., “I feel an attachment towards my own racial or ethnic heritage”) and cultural practice (i.e., “I participate in cultural practices of my own group, such as special food, music, or customs”), rated on a Likert scale from 1 = “strongly agree” to 4 = “strongly disagree.” Internal consistency, calculated using Spearman-Brown’s corrected split-half reliability, for cultural attachment and cultural practice variables was modest (.55), so results involving this measure should be interpreted cautiously.

### Data analysis

All analyses were conducted in R (v4.2.1). Code necessary to reproduce the analyses here are publicly accessible at https://osf.io/jrasx.

#### Descriptive analyses

Means, standard deviations, and bivariate correlations were computed for the final sample and separately by racial-ethnic group. Analyses of Variance (ANOVAs) tested group differences in the study variables across the three racial-ethnic groups.

#### Cross-sectional network analyses

Cross-sectional networks were constructed separately for the 3 timepoints (ages 3, 5, and 9), and within each racial-ethnic group, yielding a total of 9 networks. We used the Gaussian Graphic Model (GGM; Epskamp et al., 2018) to visualize cross-sectional associations between irritability, parenting stress (i.e., aggravation in parenting), non-violent discipline, psychological aggression, physical assault, and neglect, with cultural variables (i.e., cultural attachment and values) included to adjust for mothers’ degree of cultural belonging. The GGM estimates partial correlation coefficients (called edges) between two observed variables (called nodes), while controlling for all other variables in the network. These cross-sectional models were rerun including demographic variables (mothers’ age, income, education, relationship status) as covariates and results remained unchanged. Consequently, demographic variables were not included in the final analyses. Network comparison tests were used to compare differences in edge weights between two racial-ethnic groups (i.e., Black – White, Black – Latine or White – Latine) for all edges in the network at all timepoints (i.e., age 3, 5 and 9). Network comparison tests were also used to compare differences in edge weights between two timepoints (i.e., Age 3–5, Age 5–9, Age 3–9) in each racial-ethnic group. See Method S1 for details about network construction.

#### Longitudinal network analyses

Longitudinal models were estimated using a graphical vector-autoregression (GVAR) model with the *panelgvar*() function from the psychonetrics package (v0.10; Epskamp, 2020) for each race-ethnicity and included the irritability and parenting stress and behavior nodes. Cultural belonging variables were not included in this analysis as they were only measured at one timepoint. The panel GVAR encodes temporal dependencies as partial correlations (or edges) between the deviations from the person-wise mean in one variable at a certain timepoint and the deviations from the person-wise mean in the next timepoint while controlling for all the variables at the previous timepoint. Temporal effects are pooled across consecutive timepoints, yielding a matrix of regression coefficients that can be used to plot a directed lag-1 network (i.e., the temporal or longitudinal network), which represents the generalized temporal within-subject effects between variables (Epskamp, 2020). See Method S2 for details about network construction.

#### Comparison of specific edges in race-ethnicity longitudinal networks

We used a permutation procedure to assess the statistical differences in edge strengths between racial-ethnic groups (Black vs. White vs. Latine participants) in our longitudinal networks. See Method S3 for details about network construction.

## Results

### Descriptive results

Means, SDs and group differences of the study variables across the three timepoints, by race-ethnicity, are presented in Table 2. Bivariate correlations of the study variables are presented in Tables S2–5. At age 3, irritability scores were higher in Black than Latine participants. At age 9, irritability scores were higher in Black than Latine participants, and higher in White than Latine participants. There were significant group differences in non-violent discipline, psychological discipline, and physical assault scores between the different races-ethnicities at all three timepoints. Non-violent discipline scores (reverse-coded) followed the pattern White > Black > Latine participants at all timepoints. Psychological aggression scores showed Black > White > Latine participants at ages 3 and 9; at age 5, Black participants scored higher than both White and Latine participants. Physical assault scores showed: Black > White > Latine participants at age 3, while at ages 5 and 9, Black participants scored higher than both White and Latine participants. Neglect scores showed Black > White participants at age 3 only. Parenting stress scores showed White > Latine participants and Black > Latine participants at age 9 only.

**Table 2.** Average scores for each variable, by timepoint and participant race-ethnicity

Mean (SD)	White participants	Black participants	Latine participants	Total sample
Irritability age 3	0.64 (0.46)	0.68 (0.55)*	0.59 (0.48)*	0.65(0.51)
Irritability age 5	0.57 (0.49)	0.55 (0.52)	0.50 (0.50)	0.55 (0.51)
Irritability age 9	0.35 (0.45)*	0.33 (0.42)^	0.27 (0.40)*^	0.32 (0.42)
Parenting stress age 3	2.22 (0.57)	2.26 (0.66)	2.20 (0.69)	2.23 (0.65)
Parenting stress age 5	2.17 (0.60)	2.18 (0.69)	2.11 (0.69)	2.16 (0.67)
Parenting stress age 9	2.04 (0.63)*	2.08 (0.70)^	1.90 (0.67)*^	2.03 (0.68)
Non-violent discipline age 3	1.24 (1.09)*	1.84 (1.33)*	2.16 (1.44)*	1.77 (1.35)
Non-violent discipline age 5	1.08 (1.09)*	1.71 (1.33)*	2.01 (1.44)*	1.63 (1.30)
Non-violent discipline age 9	1.89 (1.27)*	2.41 (1.41)*	2.96 (1.57)*	2.42 (1.47)
Psychological aggression age 3	1.57 (0.91)*	1.75 (0.97)*	1.37 (0.92)*	1.60 (0.95)
Psychological aggression age 5	1.64 (0.91)*	1.86 (1.00)*^	1.57 (0.92)^	1.73 (0.99)
Psychological aggression age 9	1.43 (0.99)*	1.57 (1.09)*	1.21 (1.00)*	1.45 (1.05)
Physical assault age 3	1.10 (0.88)*	1.48 (1.06)*	0.90 (0.85)*	1.23 (0.99)
Physical assault age 5	0.89 (0.82)*	1.32 (1.00)*^	0.89 (0.91)^	1.10 (0.96)
Physical assault age 9	0.53 (0.71)*	0.90 (0.96)*^	0.59 (0.74)^	0.73 (0.87)
Neglect age 3	0.04 (0.15)*	0.07 (0.31)*	0.08 (0.36)	0.06 (0.29)
Neglect age 5	0.04 (0.17)	0.06 (0.21)	0.06 (0.21)	0.05 (0.20)
Neglect age 9	0.11 (0.31)	0.14 (0.42)	0.15 (0.37)	0.14 (0.38)

*Note.* *significant group differences, ^significant group differences.

### Cross-sectional networks

Given our research aims, we focused on interpreting paths between child irritability and parenting variables.

**Figure 1.**
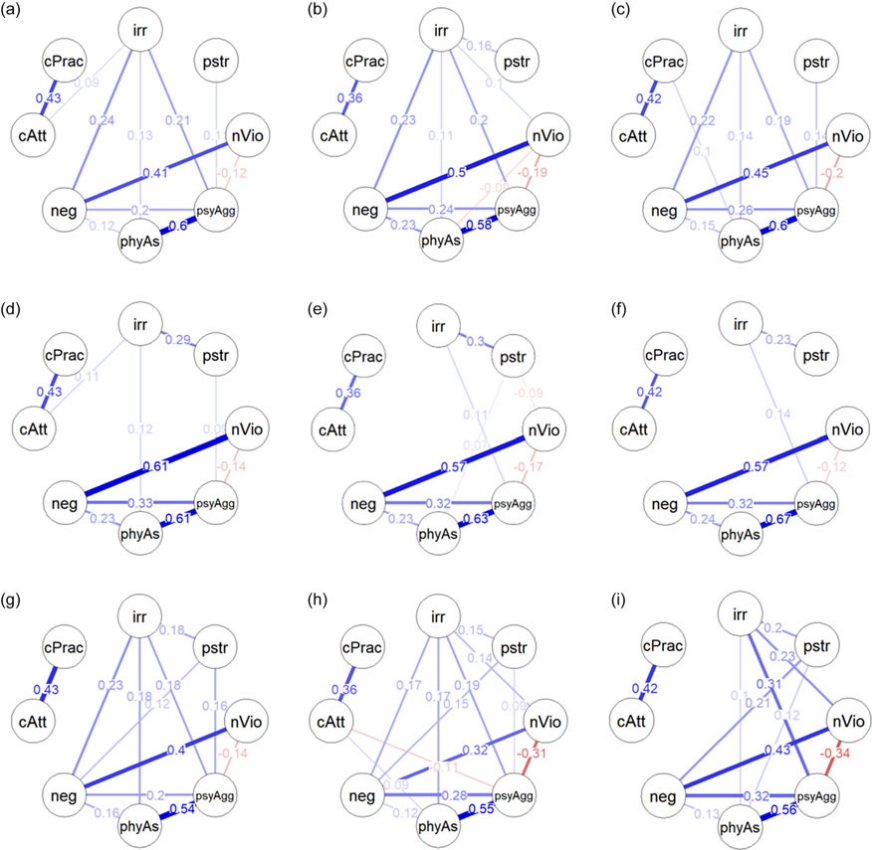
Cross-sectional network showing partial correlations between irritability and parenting variables at Age 3 for White (a), Black (b) and Latine participants (c); Age 5 for White (d), Black (e) and Latine participants (f); Age 9 for White (g), Black (h) and Latine participants (i). irr = child irritability; pstr = parenting stress; nVio = non-violent discipline; psyAgg = psychological aggression; phyAs = physical assault; neg = neglect; cAtt = mother cultural attachment; cPra = mother cultural practice. Blue edges between nodes indicate positive partial correlations, while red edges indicate negative partial correlations. The thicker the edges, the stronger the correlations between the nodes. LASSO algorithm was used to determine which edges were included in the final networks (and all significant edges are included in these networks).

Figure 1a–i presents the cross-sectional networks of all three races-ethnicities across the three timepoints. Overall, at each timepoint, the network structure was relatively similar across races-ethnicities.

At age 3 (Figure 1a–1c), child irritability was positively correlated with neglect, psychological aggression, and physical assault for all three races-ethnicities (partial correlation = 0.11–0.24). In addition, parenting stress was positively associated with child irritability (partial correlation = 0.16), and non-violent discipline was correlated with less child irritability (partial correlation = 0.10), only in Black participants. However, network comparison tests showed no significant group differences in the associations between child irritability and parenting variables at age 3 (Table 3).

**Table 3.** Network comparison tests comparing differences in edge weights for two networks at the same timepoint, for all cross-sectional networks

	B – W age3	B – L age3	W – L age 3	B – W age 5	B – L age 5	W – L age 5	B – W age 9	B – L age 9	W – L age 9
irr - pstr	0.16^	0.16^	–	–	–	–	–	–	–
irr - nVio	–	–	–	–	–	–	0.14*	0.09*	0.23**
pstr - nVio	–	–	–	–	–	–	–	–	–
irr - psyAgg	–	–	–	–	–	–	–	.12*	–
pstr - psyAgg	–	0.14*	–	–	–	–	–	–	–
nVio - psyAgg	–	–	–	0.03**	–	0.02*	0.17***	–	0.21**
irr - phyAs	–	–	–	–	–	–	–	–	–
pstr - phyAs	–	–	–	–	–	–	–	–	–
nVio - phyAs	–	0.09*	–	–	–	–	–	–	–
psyAgg - phyAs	–	–	–	–	–	–	–	–	–
irr - neg	–	–	–	–	–	–	–	0.17***	0.23***
pstr - neg	–	–	–	–	–	–	–	–	–
nVio - neg	–	–	–	–	–	–	0.08^	0.11**	–
psyAgg – neg	–	–	–	–	–	–	–	–	0.12*
phyAs - neg	–	–	–	–	–	–	–	–	–
irr - cAtt	–	–	–	–	–	–	–	–	–
pstr - cAtt	–	–	–	–	–	–	–	–	–
nVio - cAtt	–	–	–	–	–	–	–	–	–
psyAgg - cAtt	–	–	–	–	–	–	0.11**	0.11*	–
phyAs - cAtt	–	–	–	–	–	–	0.09**	0.09**	–
neg - cAtt	–	–	–	–	–	–	–	–	–
irr - cPra	–	–	–	–	–	–	–	–	–
pstr - cPra	–	–	–	–	–	–	–	–	–
nVio - cPra	–	–	–	–	–	–	–	–	–
psyAgg - cPra	–	–	–	–	–	–	–	–	–
phyAs - cPra	–	0.10**	0.10**	–	–	–	–	–	–
neg - cPra	–	–	–	–	–	–	–	–	–
cAtt - cPra	–	–	–	–	–	–	–	–	–

*Note.* Values represent absolute edge differences [AED] between groups. irr = child irritability; pstr = parenting stress; nVio = non-violent discipline; psyAgg = psychological aggression; phyAs = physical assault; neg = neglect; cAtt = mother cultural attachment; cPra = mother cultural practice. ^*p* < .10, *p < .05, **p < .01, ***p < .001, absent values = no significant differences. B – W = Black–White; B – L = Black–Latine; W – L = White–Latine.

At age 5 (Figure 1d–1f), child irritability was positively correlated with parenting stress for all races-ethnicities (0.23-0.30). In addition, psychological aggression was associated with child irritability only in Black (0.11) and Latine (0.14) participants while physical assault was associated with child irritability in White participants only (0.12). However, network comparison tests showed no significant group differences in the associations between child irritability and parenting variables at age 5 (Table 3).

At age 9 (Figure 1g–1i), child irritability was positively correlated with psychological aggression, physical assault, and parenting stress for all races-ethnicities (0.10–0.31), with the only significant group difference between Black and Latine participants in the association between irritability and psychological aggression (absolute edge differences [AED] = 0.12; Table 3). Neglect was associated with child irritability in White (0.23) and Black (.17) participants but not in Latine (0) participants, with this association being significantly different between Black and Latine participants (AED = 0.17, Table 3) and between White and Latine participants (AED = 0.23; Table 3). Non-violent discipline was correlated with less child irritability in Black (0.14) and Latine (0.23) participants but not in White (0) participants, with this association being significantly different between all racial-ethnic pairwise group comparisons (Black and White, AED = 0.14; Black and Latine, AED = 0.09; White and Latine, AED = 0.23; Table 3).

Results of network comparison tests comparing differences in edge weights between two timepoints (i.e., Age 3–5, Age 5–9, Age 3–9) in each racial-ethnic group are presented in the Supplement (Table S6).

### Longitudinal networks

**Figure 2.**
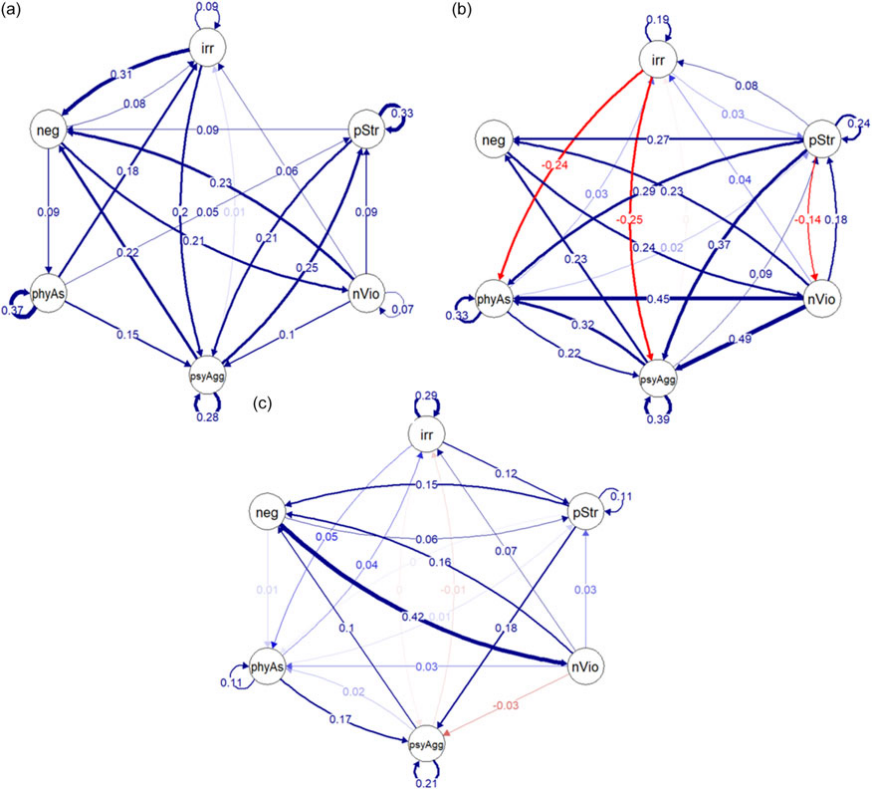
Longitudinal network showing partial correlations between irritability and parenting variables for White participants (a), Black participants (b) and Latine participants (c). Only edges (partial correlations) or autoregressive edges (correlation between a variable at a given time and the same variable at a previous timepoint) that were included at least 500 times in the 1,000 bootstrapped models are displayed. Temporal arrows represent lag-1 predictive effects, with information pooled across consecutive intervals (Age 3→5 and Age 5→9) to estimate consistent temporal effects. irr = child irritability; pstr = parenting stress; nVio = non-violent discipline; psyAgg = psychological aggression; phyAs = physical assault; neg = neglect.

The pruned longitudinal models (Figure 2a–c) showed good fit (White participants: Comparative Fit Index [CFI] = .96, Tucker–Lewis Index [TLI] = .93, Root Mean Square Error of Approximation [RMSEA] = 0.05; Black participants: CFI = 0.95, TLI = 0.93, RMSEA = 0.06; Latine participants: CFI = 0.95, TLI = 0.93, RMSEA = 0.07). The bootstrapping results (Tables S8–10) were used to exclusively plot edges that were included in at least 50% (500 out of 1,000) of the bootstrapped models, to account for the stability of the edges and to facilitate interpretation and visualization of the network results. Given our research aims, we focused on interpreting paths between child irritability and parenting variables.

In the longitudinal network model for White participants (Figure 2a), child irritability had a bidirectional positive correlation with both neglect and psychological aggression (partial correlation = .31 and.08). Physical assault and lower use of non-violent discipline both predicted increased child irritability (partial correlation = .18 and.06, respectively). Parenting stress did not directly predict child irritability. Neglect predicted reduced use of non-violent discipline (0.21), which in turn predicted increased child irritability (0.06). Child irritability and all parenting variables except neglect were stable over time, as indexed by significant autoregressive effects (i.e., correlations between the same variable across adjacent timepoints).

In the longitudinal network for Black participants (Figure 2b), child irritability was bidirectionally and positively associated with parenting stress (0.08 and 0.03). Child irritability was also bidirectionally correlated with physical assault; however, child irritability forecasted decreases in physical assault (−0.24), while physical assault forecasted increases in future child irritability (0.03). Irritability predicted decreases in psychological aggression (−0.25). Non-violent discipline also predicted decreases in child irritability (0.04). Although neglect did not directly predict child irritability, it predicted decreased non-violent discipline (0.24), which in turn predicted increased child irritability (0.04). Child irritability and all parenting variables except neglect and non-violent discipline were stable over time.

In the longitudinal network for Latine participants (Figure 2c), child irritability was bidirectionally and positively correlated with physical assault (0.04 and 0.05). Psychological aggression (−0.01) and non-violent discipline (0.07) were weakly associated with child irritability. Parenting stress did not predict child irritability. Although neglect did not directly predict child irritability, it predicted decreased non-violent discipline (0.42), which in turn predicted increased child irritability (0.07). Child irritability and all parenting variables except neglect and non-violent discipline were stable over time.

Across race-ethnicity groups, physical assault predicted future increases in child irritability while non-violent discipline predicted future decreases in child irritability. In addition, although neglect was not directly correlated with child irritability, there was a consistent indirect association with non-violent discipline for all racial-ethnic groups, i.e., neglect predicted decreased non-violent discipline, which in turn predicted increased child irritability.

#### Comparison of specific edges in race-ethnicity longitudinal networks

Comparison of specific edge strength between the three temporal networks (see Table S7) showed that four edges were significantly different (*p* < .05) between groups, none of which were related to child irritability: (1) the edge from non-violent discipline to physical assault between Black (estimate = 0.45) and White (not included in network; association was not stable and did not appear more than 50% of the time when bootstrapping) participants; (2) the edge from non-violent discipline to psychological aggression between Black (estimate = 0.45) and Latine (estimate = 0.03) participants; (3) the edge from psychological aggression to physical assault between Latine (estimate = 0.02) and White (not included in network) participants; and (4) the autoregressive edge (i.e., correlations between the same variable at adjacent timepoints) of non-violent discipline between Black (estimate = 0.07) and Latine (not included in network) participants. Only one edge involving child irritability was marginally significant (*p* = .06) between groups, i.e., the partial correlation from irritability to neglect between Black (not stable/included in the bootstrapped network) and White (estimate = 0.31) participants.

## Discussion

This is the first study to examine the link between child irritability and parenting behaviors/stress in a culturally diverse sample using a novel temporal network approach. This approach is well suited to estimating and visualizing temporal, dynamic, complex inter-relations among multiple variables while controlling for all others in the network. Results revealed bidirectional associations between parenting behaviors/stress and child irritability over ages 3, 5, and 9 in multiple racial-ethnic (Black, White, Latine) groups. These reciprocal patterns align with Sameroff’s transactional model (Sameroff, 2009) and Patterson’s parent-child coercive cycle framework (Patterson, 2002), providing further support for these developmental mechanisms. Overall, parenting behaviors and stress were associated with child irritability both concurrently and over time, with more similarities than differences in the cross-sectional and longitudinal associations between parenting and child irritability across the three racial-ethnic groups. Specifically, longitudinally, physical assault predicted increased future child irritability while non-violent discipline predicted decreased future child irritability consistently across groups. Neglect indirectly predicted increased child irritability via decreased use of non-violent discipline. Cross-sectionally, the link between child irritability and parenting was similar across groups at age 3 and age 5; the only significant group differences emerged at age 9 in the associations of child irritability with psychological aggression, neglect, and non-violent discipline.

This study demonstrated that physical assault was associated with future increases in child irritability across all investigated demographics from ages 3 to 9, aligning with evidence that parental corporal punishment predicts future irritability in children (Derella et al., 2020; Valencia et al., 2021). This pattern held despite differences in the frequency of physical assault at different timepoints (i.e., Black > White > Latine participants at age 3; Black > White participants and Black > Latine participants at ages 5 and 9). Thus, even with known racial-ethnic variation in harsh parenting (Taillieu et al., 2014) and in attitudes toward physical punishment (Lansford et al., 2004), physically aggressive parenting appears to elevate child irritability in a similar manner across various racial-ethnic groups, extending prior research (Gershoff et al., 2012). One possible mechanism is observational modeling: when caregivers use physical aggression, children may infer that aggression is an acceptable and effective way to resolve conflict (Di Giunta et al., 2020).

Non-violent discipline also consistently mitigated future irritability across racial-ethnic groups from ages 3 to 9. This aligns with existing literature that reducing negative parenting lowers the likelihood of adverse child outcomes (Sege et al., 2018). At the same time, our data and prior work indicate that non-violent discipline (e.g., time-outs, removing privileges) frequently co-occur with harsh disciplinary practices (Regalado et al., 2004). For example, a parent may offer explanations for appropriate behavior in a harsh manner or pair them with verbal threats (Lansford & Deater-Deckard, 2012). Such combinations are problematic: harsh behaviors such as verbal threats are known to increase child irritability (Derella et al., 2020; Hibbard et al., 2012; Hosokawa & Katsura, 2019) and may undermine the benefits of non-violent discipline. This underscores the need for interventions that promote positive strategies while phasing out harsh disciplinary practices. Because these patterns were broadly similar across racial-ethnic groups despite cultural variation in parenting (Bornstein, 2012; Coll et al., 1996), our findings support culturally wide relevance of non-violent discipline for fostering healthy development.

Non-violent discipline also mediated the pathway between child irritability and neglect for all racial-ethnic groups. Specifically, higher levels of neglect predicted lower use of non-violent discipline, which in turn was associated with greater child irritability. Given that neglect reflects a lack of parental involvement and supervision (Ravi et al., 2022), this finding suggests that neglectful parents may engage less frequently in proactive or non-violent forms of discipline. The presence of this indirect pathway across all racial and ethnic groups, despite the absence of a direct association between neglect and child irritability, underscores the broad role of neglect in shaping parenting behaviors. These results highlight the importance of addressing neglect within parenting interventions, particularly as it relates to the reduced use of non-violent disciplinary strategies.

We note that no significant group differences emerged in longitudinal associations of child irritability with neglect, psychological aggression, and parenting stress across racial-ethnic groups. Deviating from Coll’s integrative model (1996) suggesting that there may be differences in child competencies due to variations in culture and familial values, our results instead suggest that there was more cultural commonality than specificity in the associations between parenting and child irritability across racial-ethnic groups.

Cross-sectionally at age 3 and age 5, the link between child irritability and parenting was also similar across groups; the only significant group differences emerged at age 9 in the associations of child irritability and psychological aggression (between Black–Latine networks), neglect (between Black–Latine and White–Latine networks), and non-violent discipline (between all three comparison networks). A developmental lens may help explain why racial-ethnic differences appear in late rather than early childhood. In early childhood, children rely heavily on caregivers for emotional regulation, direct supervision, and daily care, whereas in middle and late childhood, they have greater autonomy and more frequent interactions with peers and teachers (Rubin et al., 2006). As children encounter greater diversity in beliefs and practices in school and community settings, parents may adjust their approaches to discipline and emotion socialization in response to children’s changing values (Knafo & Galansky, 2008). Taken together, these findings align with Coll’s emphasis on sociocultural context (1996), Bornstein’s distinction between the form and function of parenting (2012), and Lansford’s notion of cultural normativeness (2021), indicating that cultural differences in the associations between parenting behaviors and irritability emerge in late childhood as children’s expanding social environments make cultural norms more influential. These findings suggest that intervention programs, such as those that emphasize non-violent discipline for example, may need to be developmentally timed.

This study has several strengths including the use of an innovative temporal network analysis with large-scale longitudinal data to elucidate the directional associations between child irritability and parenting behaviors/stress in a culturally diverse sample. This novel approach enables us to visualize the bidirectional links of all variables in the network, supporting Sameroff’s transactional framework (2009), and allows for the identification of possible indirect pathways that traditional approaches requiring pre-specified directional hypotheses might miss. The findings lend support for universal best parenting practices, such as non-violent discipline strategies recommended by the American Academy of Pediatrics (Sege et al., 2018). Cross-cultural consistency in these associations suggests that evidence-based interventions may be broadly applicable across diverse cultural settings, with local adaptation accounting for variations in adaptive culture and family values, as theorized by Coll et al. (1996). This may have important implications for contexts with limited resources or high adaptation costs, enabling more efficient and equitable support for families from varied backgrounds.

Notable limitations include the lack of measures directly probing cultural factors, values, and beliefs relevant to each racial-ethnic group or stress associated with racial discrimination affecting minority groups. Thus, while our study provides preliminary evidence of similarities and differences in the temporal associations between child irritability and parenting behaviors/stress across race-ethnicity groups, future research is imperative to uncover specific underlying factors. Although the FFCWS sample is valuable for studying marginalized and minority populations, it is US-based and results of this study may have limited generalizability to the broader population including those outside the US and other minoritized groups (e.g., Asian, Native American or mixed-race populations) not included in the study. In addition, the oversampling of unmarried mothers by a 3:1 ratio and low socioeconomic status (54.6% of participants had a household income of less than $25,000) may have obscured differences between racial groups, as these factors may be associated with greater financial hardship, stress, and child-rearing challenges across groups. Future research should investigate a variety of socioeconomic and familial statuses to gain a fuller understanding of their impact on irritability and parenting. Overgeneralizations to the Latine population should also be made cautiously, given the group’s heterogeneity in heritage and immigration statuses. In addition, the data relied mostly on maternal reports only and did not include paternal or child reports. We also found low reliability coefficients for the parenting behavior subscales, although this is consistent with previous reports (Guterman et al., 2009; Spencer et al., 2021). As a result, scale-level interpretations should be made with caution due to these lower internal consistencies in the parenting variables. Cultural attachment and cultural practice variables also had low internal consistency, and although this was our best approximation of the cultural values and beliefs relevant to each group due to the lack of direct measures, further research is needed to investigate the role of specific cultural factors. Finally, while longitudinal data was used, the temporal network analysis encodes directional partial correlations between lagged and non-lagged variables, and therefore causality cannot be inferred.

The present study demonstrates longitudinal, bidirectional associations between child irritability and parenting behaviors and stress using a novel temporal network analysis approach. Across racial-ethnic groups, we found more similarities than differences in these associations, a finding that nuances prevailing assumptions about ethnic-racial differences. Future work is needed to elucidate factors relevant to each racial-ethnic group and cultures that contribute to these similarities and differences. Although our results show that broadly, parenting behaviors may have similar effects on child irritability across groups, their interpretations and meaning can differ by cultural context. Therefore, implementing these findings requires cultural sensitivity to underlying values and beliefs. By investigating how parenting behaviors relate to child irritability and how these associations vary by cultures, these findings can challenge biased assumptions about ethnic-racial differences in parenting and inform interventions that promote positive child outcomes and well-being across diverse cultural backgrounds.

## Supporting information

10.1017/S095457942610145X.sm001 Lim et al. supplementary material Lim et al. supplementary material

## Data Availability

Data from this study is available at https://ffcws.princeton.edu/data-and-documentation/getting-started. Code and materials necessary to reproduce the analyses here are publicly accessible at https://osf.io/jrasx.

## Supplementary material

The supplementary material for this article can be found at https://doi.org/10.1017/S095457942610145X.

## Preregistration statement

Analyses were not preregistered as this study relied on a pre-existing dataset that had been collected prior to the development of the research questions, therefore prospective preregistration was not feasible.
