# Supplementary material for: Parenting practices and child irritability across diverse racial–ethnic backgrounds: A temporal network analysis
Source: Dev Psychopathol. 2026 May 5:1–12. Online ahead of print. doi: 10.1017/S095457942610145X (PMC13222754; doi:10.1017/S095457942610145X)
Supplement: Lim et al. supplementary material [file S095457942610145Xsup001.docx]

**Parenting Practices and Child Irritability across Diverse Racial-Ethnic Backgrounds: A Temporal Network Analysis (SUPPLEMENT)**

**Method S1**

*Cross-sectional network analyses*

Cross-sectional networks were constructed separately for the 3 timepoints, with separate networks for each race-ethnicity, yielding a total of 9 networks. The Gaussian Graphic Model (Epskamp, Waldorp, et al., 2018) was used to measure the cross-sectional associations between irritability, parenting stress (i.e., aggravation in parenting), non-violent discipline, psychological aggression, physical assault, and neglect, with cultural variables (i.e., cultural attachment and values) included in order to explore if they impact parenting, given known associations between cultural variables and parenting practices. The network model estimates partial correlation coefficients (also called edges) between two observed variables, represented as nodes, while controlling for all other variables in the network. Although the variables used in the network were averaged scores, they did not satisfy the criteria for continuous variables and were therefore treated as ordinal variables. To account for the non-normality of the data, we applied a nonparanormal transformation (Liu et al., 2009) using the huge package (Zhao, Liu, Roeder, Lafferty, & Wasserman, 2012; v1.3.5). Graphical least absolute shrinkage and selection operator (LASSO) regularization was implemented to limit spurious edges (Friedman et al., 2008) using the ggmModSelect function from the bootnet package (Epskamp, Borsboom, & Fried, 2018; v1.5) with a rank-order transformation (Spearman correlations). The edge strengths and node centrality indices (strength, closeness, and betweenness) of the networks were calculated to characterize the connectivity of the network structure, and bootstrapping (Epskamp, Borsboom, et al., 2018) was used to assess the robustness of the parameters. To create the network graphs, the *bootnet* (v1.5), *psychonetrics* (v0.10), and *qgraph* (v1.9.2) R packages were used. The node layout in each network was fixed using a circle layout.

**Method S2**

*Longitudinal network analyses*

Longitudinal models were estimated using a graphical vector-autoregression (GVAR) model with the *panelgvar*() function from the psychonetrics package (Epskamp, 2020; v0.10) for each race-ethnicity and included the irritability and parenting stress and behavior nodes. Cultural belonging variables were not included in this analysis as they were only measured at one timepoint. The panel GVAR encodes temporal dependencies as partial correlations (or edges) between the deviations from the person-wise mean in one variable at a certain timepoint and the deviations from the person-wise mean in the next timepoint while controlling for all the variables at the previous timepoint. This yields a matrix of regression coefficients that can be used to plot a directed lag-1 network (i.e., the temporal or longitudinal network), which represents the generalized temporal within-subject effects between variables (Epskamp, 2020). The GVAR model assumes that variables are stable over time, so all variables were detrended before computing the networks. The variables were also treated as ordinal, consistent with the cross-sectional networks. Therefore, the GVAR model was fitted using Unweighted Least Squares estimation. Confirmatory Fit Index (CFI), Tucker-Lewis Index (TLI), and the Root Mean Squared Error of Approximation (RMSEA) were used to assess model fit. Values of >.95 on the CFI and TLI and values of < .05 on the RMSEA indicated a good fit of the model (Hooper et al., 2007). To increase robustness against spurious edges, we implemented the “stepup” pruning method, which removed the edges that did not meet the significance level of α = .05 and then re-estimated the model with these edges set to zero. We also assessed the robustness of the results by evaluating how often each edge was included in the 1,000 bootstrapped models and by including edges that appeared in over 50% of the bootstraps, following prior work (Bellaert et al., 2023). In accordance with our research aims, we focus on interpreting paths between child irritability and other variables for both cross-sectional and longitudinal networks.

**Method S3**

We used a permutation procedure to test the significant differences (considered as p < .05) in edge strengths between race-ethnicity groups (Black vs. White vs. Latine participants) in our longitudinal networks. The procedure was adapted from Klippel and colleagues (Beijer-Klippel et al., 2017) and conducted as follows. First, we fitted a model with the actual data and computed the edge strengths. Second, we calculated group differences in the edge strengths (i.e., Δ edge strength) as follows: edge strengths of White participants were subtracted from edge strengths of Black participants, edge strengths of Latine participants were subtracted from edge strengths of Black participants, and edge strengths of Latine participants were subtracted from edge strengths of White participants. Third, we randomly reshuffled group labels (Black, White, and Latine) between subjects, fitted the models, and computed each Δ edge strength. We repeated this procedure 10,000 times to obtain the permutation distributions of the size of the group differences under the null hypothesis. We compared the size of the observed Δ edge strength to the permutation distributions of the Δ edge strengths, to determine the level of significance of the group differences. The p-value was calculated by counting the number of times that a group difference for a particular edge under the permuted data was as great or greater than the one observed with the actual data and dividing this count by the total number of tests. By doubling these ratios, we obtained the two-sided p-values. This analysis showed the edges that differed significantly (two-sided p < .05) between groups as well as the difference in strengths of those edges. This permutation-based test was conducted on a cluster computer composed of 17 nodes with two Intel Skylake 16-cores Xeon 6142 processors each and 192GB of RAM using parallel processing.

**RESULTS**

**Table S6** shows differences in associations between irritability and parenting behavior for a given group at two timepoints. For White (Age 3 and 5, AED = .289; Age 3 and 9, AED = .180) and Latine (Age 3 and 5, AED = .223; Age 3 and 9, AED = .198) participants, the association between child irritability and parenting stress differed significantly at age 3, but was similar at ages 5 and 9. In contrast, for Black (Age 3 and 5, AED = .141; Age 5 and 9, AED = .145) participants, this association differed significantly at age 5, but was similar at ages 3 and 9. In addition, the association between child irritability and neglect differed significantly at age 5 for White (Age 3 and 5, AED = .244; Age 5 and 9, AED = .227) and Black (Age 3 and 5, AED = .230; Age 5 and 9, AED = .169) participants, but significantly differed at age 3 for Latine (Age 3 and 5, AED = .221; Age 3 and 9, AED = .221) participants, while remaining similar at the other timepoints. There were no further clear patterns with the associations between child irritability and other parenting behaviors.

**References**

Beijer-Klippel, A., Viechtbauer, W., Reininghaus, U., Wigman, J., van Borkulo, C., MERGE, Myin-Germeys, I., & Wichers, M. (2017). The Cascade of Stress: A Network Approach to Explore Differential Dynamics in Populations Varying in Risk for Psychosis. *Schizophrenia Bulletin*, *44*. https://doi.org/10.1093/schbul/sbx037

Bellaert, N., Morreale, K., & Tseng, W.-L. (2023). Peer functioning difficulties may exacerbate symptoms of attention-deficit/hyperactivity disorder and irritability over time: a temporal network analysis. *Journal of Child Psychology and Psychiatry*, *n/a*(n/a). https://doi.org/https://doi.org/10.1111/jcpp.13911

Epskamp, S. (2020). Psychometric network models from time-series and panel data. *Psychometrika*, *85*(1), 206–231. https://doi.org/10.1007/s11336-020-09697-3

Epskamp, S., Borsboom, D., & Fried, E. I. (2018). Estimating psychological networks and their accuracy: A tutorial paper. *Behavior Research Methods*, *50*(1), 195–212. https://doi.org/10.3758/s13428-017-0862-1

Epskamp, S., Waldorp, L. J., Mõttus, R., & Borsboom, D. (2018). The Gaussian Graphical Model in Cross-Sectional and Time-Series Data. *Multivariate Behavioral Research*, *53*(4), 453–480. https://doi.org/10.1080/00273171.2018.1454823

Friedman, J., Hastie, T., & Tibshirani, R. (2008). Sparse inverse covariance estimation with the graphical lasso. *Biostatistics*, *9*(3), 432–441. https://doi.org/10.1093/biostatistics/kxm045

Hooper, D., Coughlan, J., & Mullen, M. (2007). Structural Equation Modeling: Guidelines for Determining Model Fit. *The Electronic Journal of Business Research Methods*, *6*.

Liu, H., Lafferty, J., Wasserman, L., & Wainwright, M. J. (2009). The Nonparanormal: Semiparametric Estimation of High Dimensional Undirected Graphs. In *Journal of Machine Learning Research* (Vol. 10).

Zhao, T., Liu, H., Roeder, K., Lafferty, J., & Wasserman, L. (2012). *The huge Package for High-dimensional Undirected Graph Estimation in R*.

**Table S1.** Socio-demographic characteristics of the analytic and excluded sample.

| Socio-demographic variable | | Included Sample  (N = 2408) | Excluded Sample  (N = 2490) | Comparison |  |
| --- | --- | --- | --- | --- | --- |
|  | Mean | | |  |  |
| Mother Age at Child Age 1 | | 25.22 (6.04) | 25.32 (6.04) | *t* = -0.59, *p* = .56 |  |
|  | n (%) | | |  |  |
| Child Gender | |  |  | χ²(1) = 1.49, *p* = .22 |  |
| Male  Female  No answer | | 1235 (51.29)  1173 (48.71)  0 (0) | 1321 (53.05)  1168 (46.91)  1 (.04) |  |  |
| Mother Race/Ethnicity | | |  |  | χ²(2) = 39.87, *p* < .001 (comparing only groups retained for analysis) |
| White  Black  Latine  Other racial-ethnic group / NA | | | 614  1167  627  0 (0) | 416 (16.71)  1159 (46.55)  709 (28.47)  206 (8.27) |  |
| Mother Relationship Status | |  |  | χ²(4) = 223.21, *p* < .001 |  |
| Married  Cohabitation  Visiting  Friends  Hardly Talk, Never Talk or Father Unknown  No answer | | 666 (27.65)  986 (40.95)  595 (24.71)  94 (3.90)  64 (2.66)  0 (0) | 521 (20.92)  797 (32.01)  679 (27.27)  199 (7.99)  292 (11.73)  2 (0.08) |  |  |
| Mother Education | |  |  | χ²(3) = 34.77, *p* < .001 |  |
| Less than High School  High School  Some College or Technical/Trade School  College or Graduate/ Professional School  No answer | | 745 (30.94)  741 (30.77)  627 (26.04)  292 (12.13)  3 (.12) | 954 (38.31)  739 (29.68)  562 (22.57)  232 (9.32)  3 (.12) |  |  |
| Mother Household Income ($) | |  |  | χ²(3) = 32.52, *p* < .001 |  |
| < 25,000  25,000-50,000  50,000-75,000  >75,000 | | 1237 (54.65)  668 (26.79)  302 (11.05)  201 (8.35) | 1467 (58.92)  626 (25.14)  232 (9.32)  164 (6.59) |  |  |

**Table S2**

*Means, standard deviations, and correlations with confidence intervals for all participants at all timepoints*

| Variable | *M* | *SD* | 1 | 2 | 3 | 4 | 5 | 6 | 7 | 8 | 9 | 10 | 11 | 12 | 13 | 14 | 15 | 16 | 17 |
| --- | --- | --- | --- | --- | --- | --- | --- | --- | --- | --- | --- | --- | --- | --- | --- | --- | --- | --- | --- |
|  |  |  |  |  |  |  |  |  |  |  |  |  |  |  |  |  |  |  |  |
| 1. IRR_AGE3 | .65 | .51 |  |  |  |  |  |  |  |  |  |  |  |  |  |  |  |  |  |
|  |  |  |  |  |  |  |  |  |  |  |  |  |  |  |  |  |  |  |  |
| 2. PSTR_AGE3 | 2.23 | .65 | .17** |  |  |  |  |  |  |  |  |  |  |  |  |  |  |  |  |
|  |  |  | [.13, .21] |  |  |  |  |  |  |  |  |  |  |  |  |  |  |  |  |
|  |  |  |  |  |  |  |  |  |  |  |  |  |  |  |  |  |  |  |  |
| 3. NVIO_AGE3 | 1.77 | 1.35 | -.18** | -.07** |  |  |  |  |  |  |  |  |  |  |  |  |  |  |  |
|  |  |  | [-.22, -.13] | [-.12, -.03] |  |  |  |  |  |  |  |  |  |  |  |  |  |  |  |
|  |  |  |  |  |  |  |  |  |  |  |  |  |  |  |  |  |  |  |  |
| 4. PSYAGG_AGE3 | 1.60 | .95 | .30** | .19** | -.40** |  |  |  |  |  |  |  |  |  |  |  |  |  |  |
|  |  |  | [.26, .34] | [.15, .23] | [-.43, -.36] |  |  |  |  |  |  |  |  |  |  |  |  |  |  |
|  |  |  |  |  |  |  |  |  |  |  |  |  |  |  |  |  |  |  |  |
| 5. PHYAS_AGE3 | 1.23 | .99 | .25** | .15** | -.36** | .62** |  |  |  |  |  |  |  |  |  |  |  |  |  |
|  |  |  | [.21, .29] | [.11, .19] | [-.40, -.33] | [.60, .65] |  |  |  |  |  |  |  |  |  |  |  |  |  |
|  |  |  |  |  |  |  |  |  |  |  |  |  |  |  |  |  |  |  |  |
| 6. NEG_AGE3 | .06 | .29 | .08** | .12** | -.02 | .11** | .10** |  |  |  |  |  |  |  |  |  |  |  |  |
|  |  |  | [.04, .13] | [.07, .16] | [-.06, .02] | [.07, .15] | [.05, .14] |  |  |  |  |  |  |  |  |  |  |  |  |
|  |  |  |  |  |  |  |  |  |  |  |  |  |  |  |  |  |  |  |  |
| 7. IRR_AGE5 | .55 | .51 | .45** | .16** | -.04 | .16** | .14** | .06** |  |  |  |  |  |  |  |  |  |  |  |
|  |  |  | [.41, .48] | [.11, .20] | [-.09, .00] | [.11, .20] | [.10, .19] | [.02, .11] |  |  |  |  |  |  |  |  |  |  |  |
|  |  |  |  |  |  |  |  |  |  |  |  |  |  |  |  |  |  |  |  |
| 8. PSTR_AGE5 | 2.16 | .67 | .16** | .57** | -.08** | .17** | .14** | .09** | .27** |  |  |  |  |  |  |  |  |  |  |
|  |  |  | [.11, .20] | [.54, .60] | [-.12, -.04] | [.12, .21] | [.09, .18] | [.05, .13] | [.23, .31] |  |  |  |  |  |  |  |  |  |  |
|  |  |  |  |  |  |  |  |  |  |  |  |  |  |  |  |  |  |  |  |
| 9. NVIO_AGE5 | 1.63 | 1.30 | -.15** | -.07** | .47** | -.23** | -.22** | -.03 | -.16** | -.13** |  |  |  |  |  |  |  |  |  |
|  |  |  | [-.19, -.10] | [-.12, -.03] | [.43, .50] | [-.27, -.18] | [-.27, -.18] | [-.07, .02] | [-.21, -.12] | [-.17, -.08] |  |  |  |  |  |  |  |  |  |
|  |  |  |  |  |  |  |  |  |  |  |  |  |  |  |  |  |  |  |  |
| 10. PSYAGG_AGE5 | 1.73 | .99 | .23** | .18** | -.23** | .51** | .38** | .09** | .22** | .20** | -.41** |  |  |  |  |  |  |  |  |
|  |  |  | [.18, .27] | [.14, .23] | [-.27, -.18] | [.48, .55] | [.34, .42] | [.05, .14] | [.17, .26] | [.16, .25] | [-.44, -.37] |  |  |  |  |  |  |  |  |
|  |  |  |  |  |  |  |  |  |  |  |  |  |  |  |  |  |  |  |  |
| 11. PHYAS_AGE5 | 1.10 | .96 | .19** | .15** | -.19** | .39** | .54** | .13** | .18** | .17** | -.35** | .60** |  |  |  |  |  |  |  |
|  |  |  | [.14, .23] | [.11, .20] | [-.24, -.15] | [.35, .43] | [.51, .57] | [.08, .18] | [.13, .22] | [.13, .22] | [-.39, -.31] | [.57, .63] |  |  |  |  |  |  |  |
|  |  |  |  |  |  |  |  |  |  |  |  |  |  |  |  |  |  |  |  |
| 12. NEG_AGE5 | .05 | .20 | .09** | .11** | .02 | .07** | .08** | .14** | .08** | .13** | -.01 | .14** | .11** |  |  |  |  |  |  |
|  |  |  | [.04, .13] | [.07, .16] | [-.02, .07] | [.02, .12] | [.03, .13] | [.09, .18] | [.03, .12] | [.09, .18] | [-.06, .03] | [.09, .18] | [.06, .15] |  |  |  |  |  |  |
|  |  |  |  |  |  |  |  |  |  |  |  |  |  |  |  |  |  |  |  |
| 13. IRR_AGE9 | .32 | .42 | .33** | .11** | -.09** | .11** | .10** | .03 | .41** | .18** | -.15** | .21** | .20** | .06* |  |  |  |  |  |
|  |  |  | [.29, .37] | [.07, .15] | [-.14, -.05] | [.06, .15] | [.05, .15] | [-.02, .07] | [.37, .45] | [.14, .22] | [-.20, -.10] | [.16, .25] | [.15, .24] | [.01, .10] |  |  |  |  |  |
|  |  |  |  |  |  |  |  |  |  |  |  |  |  |  |  |  |  |  |  |
| 14. PSTR_AGE9 | 2.03 | .68 | .13** | .41** | -.10** | .15** | .16** | .05* | .16** | .48** | -.12** | .20** | .20** | .10** | .22** |  |  |  |  |
|  |  |  | [.09, .18] | [.38, .45] | [-.14, -.05] | [.11, .20] | [.11, .20] | [.00, .09] | [.11, .20] | [.45, .52] | [-.17, -.07] | [.15, .24] | [.16, .25] | [.05, .14] | [.18, .26] |  |  |  |  |
|  |  |  |  |  |  |  |  |  |  |  |  |  |  |  |  |  |  |  |  |
| 15. NVIO_AGE9 | 2.42 | 1.47 | -.13** | -.09** | .44** | -.24** | -.26** | -.03 | -.15** | -.07** | .50** | -.31** | -.28** | -.01 | -.28** | -.17** |  |  |  |
|  |  |  | [-.18, -.09] | [-.13, -.04] | [.40, .48] | [-.29, -.20] | [-.31, -.22] | [-.07, .02] | [-.19, -.10] | [-.12, -.03] | [.47, .54] | [-.35, -.27] | [-.33, -.24] | [-.06, .04] | [-.31, -.24] | [-.21, -.13] |  |  |  |
|  |  |  |  |  |  |  |  |  |  |  |  |  |  |  |  |  |  |  |  |
| 16. PSYAGG_AGE9 | 1.45 | 1.05 | .17** | .16** | -.22** | .40** | .34** | .02 | .19** | .16** | -.28** | .50** | .40** | .09** | .33** | .23** | -.55** |  |  |
|  |  |  | [.12, .21] | [.12, .20] | [-.26, -.18] | [.36, .44] | [.30, .38] | [-.02, .07] | [.15, .23] | [.12, .20] | [-.32, -.23] | [.46, .53] | [.36, .44] | [.04, .13] | [.29, .36] | [.19, .27] | [-.58, -.52] |  |  |
|  |  |  |  |  |  |  |  |  |  |  |  |  |  |  |  |  |  |  |  |
| 17. PHYAS_AGE9 | .73 | .87 | .17** | .13** | -.15** | .25** | .40** | .06** | .15** | .13** | -.19** | .32** | .48** | .06* | .28** | .19** | -.44** | .60** |  |
|  |  |  | [.12, .21] | [.09, .17] | [-.19, -.10] | [.20, .29] | [.36, .44] | [.02, .11] | [.10, .19] | [.09, .17] | [-.24, -.15] | [.27, .36] | [.45, .52] | [.01, .11] | [.24, .32] | [.15, .24] | [-.47, -.40] | [.58, .63] |  |
|  |  |  |  |  |  |  |  |  |  |  |  |  |  |  |  |  |  |  |  |
| 18. NEG_AGE9 | .14 | .38 | .10** | .10** | -.03 | .09** | .09** | .10** | .06** | .11** | -.03 | .12** | .10** | .19** | .14** | .14** | -.08** | .23** | .21** |
|  |  |  | [.05, .14] | [.06, .14] | [-.07, .02] | [.04, .13] | [.04, .13] | [.06, .15] | [.02, .11] | [.07, .15] | [-.08, .02] | [.07, .16] | [.05, .14] | [.15, .24] | [.10, .18] | [.10, .18] | [-.13, -.04] | [.19, .27] | [.16, .25] |
|  |  |  |  |  |  |  |  |  |  |  |  |  |  |  |  |  |  |  |  |

*Note.* *M* and *SD* are used to represent mean and standard deviation, respectively. Values in square brackets indicate the 95% confidence interval for each correlation. The confidence interval is a plausible range of population correlations that could have caused the sample correlation (Cumming, 2014). * indicates *p* < .05. ** indicates *p* < .01. IRR = child irritability, PSTR = parenting stress, NVIO = non-violent discipline, PSYAGG = psychological aggression, PHYAS = physical assault, NEG = neglect.

**Table S3**

*Means, standard deviations, and correlations with confidence intervals for White participants at all timepoints*

| Variable | *M* | *SD* | 1 | 2 | 3 | 4 | 5 | 6 | 7 | 8 | 9 | 10 | 11 | 12 | 13 | 14 | 15 | 16 | 17 |
| --- | --- | --- | --- | --- | --- | --- | --- | --- | --- | --- | --- | --- | --- | --- | --- | --- | --- | --- | --- |
|  |  |  |  |  |  |  |  |  |  |  |  |  |  |  |  |  |  |  |  |
| 1. IRR_AGE3 | .64 | .46 |  |  |  |  |  |  |  |  |  |  |  |  |  |  |  |  |  |
|  |  |  |  |  |  |  |  |  |  |  |  |  |  |  |  |  |  |  |  |
| 2. PSTR_AGE3 | 2.22 | .57 | .14** |  |  |  |  |  |  |  |  |  |  |  |  |  |  |  |  |
|  |  |  | [.05, .22] |  |  |  |  |  |  |  |  |  |  |  |  |  |  |  |  |
|  |  |  |  |  |  |  |  |  |  |  |  |  |  |  |  |  |  |  |  |
| 3. NVIO_AGE3 | 1.24 | 1.09 | -.19** | -.09* |  |  |  |  |  |  |  |  |  |  |  |  |  |  |  |
|  |  |  | [-.27, -.11] | [-.18, -.01] |  |  |  |  |  |  |  |  |  |  |  |  |  |  |  |
|  |  |  |  |  |  |  |  |  |  |  |  |  |  |  |  |  |  |  |  |
| 4. PSYAGG_AGE3 | 1.57 | .91 | .34** | .25** | -.31** |  |  |  |  |  |  |  |  |  |  |  |  |  |  |
|  |  |  | [.26, .41] | [.17, .33] | [-.38, -.23] |  |  |  |  |  |  |  |  |  |  |  |  |  |  |
|  |  |  |  |  |  |  |  |  |  |  |  |  |  |  |  |  |  |  |  |
| 5. PHYAS_AGE3 | 1.10 | .88 | .27** | .18** | -.31** | .60** |  |  |  |  |  |  |  |  |  |  |  |  |  |
|  |  |  | [.19, .35] | [.10, .26] | [-.38, -.23] | [.55, .66] |  |  |  |  |  |  |  |  |  |  |  |  |  |
|  |  |  |  |  |  |  |  |  |  |  |  |  |  |  |  |  |  |  |  |
| 6. NEG_AGE3 | .04 | .15 | .09* | .25** | -.02 | .14** | .10* |  |  |  |  |  |  |  |  |  |  |  |  |
|  |  |  | [.00, .17] | [.17, .33] | [-.11, .06] | [.06, .22] | [.01, .18] |  |  |  |  |  |  |  |  |  |  |  |  |
|  |  |  |  |  |  |  |  |  |  |  |  |  |  |  |  |  |  |  |  |
| 7. IRR_AGE5 | .57 | .49 | .51** | .16** | -.08 | .20** | .19** | .16** |  |  |  |  |  |  |  |  |  |  |  |
|  |  |  | [.44, .57] | [.08, .24] | [-.17, .01] | [.11, .28] | [.10, .27] | [.07, .24] |  |  |  |  |  |  |  |  |  |  |  |
|  |  |  |  |  |  |  |  |  |  |  |  |  |  |  |  |  |  |  |  |
| 8. PSTR_AGE5 | 2.17 | .60 | .13** | .61** | -.14** | .24** | .20** | .23** | .26** |  |  |  |  |  |  |  |  |  |  |
|  |  |  | [.04, .21] | [.56, .66] | [-.23, -.06] | [.16, .32] | [.12, .28] | [.15, .31] | [.19, .34] |  |  |  |  |  |  |  |  |  |  |
|  |  |  |  |  |  |  |  |  |  |  |  |  |  |  |  |  |  |  |  |
| 9. NVIO_AGE5 | 1.08 | 1.02 | -.11* | -.10* | .50** | -.16** | -.19** | -.04 | -.16** | -.14** |  |  |  |  |  |  |  |  |  |
|  |  |  | [-.20, -.02] | [-.19, -.01] | [.42, .56] | [-.25, -.07] | [-.28, -.10] | [-.14, .05] | [-.25, -.07] | [-.22, -.05] |  |  |  |  |  |  |  |  |  |
|  |  |  |  |  |  |  |  |  |  |  |  |  |  |  |  |  |  |  |  |
| 10. PSYAGG_AGE5 | 1.64 | .92 | .23** | .20** | -.16** | .54** | .37** | .10* | .24** | .26** | -.34** |  |  |  |  |  |  |  |  |
|  |  |  | [.14, .32] | [.11, .28] | [-.25, -.07] | [.47, .60] | [.29, .45] | [.01, .20] | [.15, .33] | [.17, .34] | [-.41, -.26] |  |  |  |  |  |  |  |  |
|  |  |  |  |  |  |  |  |  |  |  |  |  |  |  |  |  |  |  |  |
| 11. PHYAS_AGE5 | .88 | .82 | .18** | .16** | -.23** | .43** | .64** | .09 | .21** | .16** | -.29** | .55** |  |  |  |  |  |  |  |
|  |  |  | [.09, .27] | [.07, .24] | [-.31, -.14] | [.35, .50] | [.58, .69] | [-.01, .18] | [.12, .30] | [.08, .25] | [-.37, -.21] | [.48, .61] |  |  |  |  |  |  |  |
|  |  |  |  |  |  |  |  |  |  |  |  |  |  |  |  |  |  |  |  |
| 12. NEG_AGE5 | .04 | .17 | .06 | .11* | -.02 | .04 | .06 | .16** | .09 | .12** | -.01 | .10* | .03 |  |  |  |  |  |  |
|  |  |  | [-.03, .15] | [.02, .19] | [-.12, .07] | [-.05, .14] | [-.03, .15] | [.07, .25] | [-.00, .18] | [.03, .21] | [-.10, .08] | [.01, .19] | [-.06, .11] |  |  |  |  |  |  |
|  |  |  |  |  |  |  |  |  |  |  |  |  |  |  |  |  |  |  |  |
| 13. IRR_AGE9 | .35 | .45 | .35** | .14** | -.08 | .10* | .08 | .08 | .43** | .20** | -.10* | .22** | .21** | .01 |  |  |  |  |  |
|  |  |  | [.27, .43] | [.05, .22] | [-.17, .01] | [.00, .18] | [-.01, .17] | [-.01, .17] | [.35, .50] | [.11, .28] | [-.20, -.01] | [.13, .31] | [.12, .30] | [-.08, .11] |  |  |  |  |  |
|  |  |  |  |  |  |  |  |  |  |  |  |  |  |  |  |  |  |  |  |
| 14. PSTR_AGE9 | 2.04 | .62 | .17** | .48** | -.17** | .18** | .22** | .17** | .16** | .53** | -.17** | .28** | .25** | .17** | .30** |  |  |  |  |
|  |  |  | [.08, .25] | [.42, .55] | [-.26, -.09] | [.09, .26] | [.13, .30] | [.08, .25] | [.07, .24] | [.47, .59] | [-.26, -.08] | [.19, .36] | [.16, .34] | [.08, .26] | [.22, .37] |  |  |  |  |
|  |  |  |  |  |  |  |  |  |  |  |  |  |  |  |  |  |  |  |  |
| 15. NVIO_AGE9 | 1.89 | 1.27 | -.13** | -.13** | .34** | -.16** | -.18** | -.11* | -.19** | -.10* | .51** | -.29** | -.27** | -.01 | -.31** | -.24** |  |  |  |
|  |  |  | [-.22, -.04] | [-.22, -.05] | [.26, .42] | [-.24, -.07] | [-.27, -.10] | [-.20, -.02] | [-.28, -.11] | [-.18, -.01] | [.44, .58] | [-.37, -.20] | [-.36, -.18] | [-.11, .08] | [-.38, -.23] | [-.32, -.16] |  |  |  |
|  |  |  |  |  |  |  |  |  |  |  |  |  |  |  |  |  |  |  |  |
| 16. PSYAGG_AGE9 | 1.43 | .99 | .14** | .18** | -.14** | .39** | .37** | .08 | .19** | .18** | -.20** | .55** | .46** | .05 | .34** | .31** | -.44** |  |  |
|  |  |  | [.05, .23] | [.09, .26] | [-.23, -.05] | [.31, .47] | [.29, .44] | [-.01, .17] | [.10, .28] | [.10, .27] | [-.29, -.11] | [.48, .61] | [.38, .53] | [-.05, .14] | [.26, .41] | [.23, .39] | [-.51, -.37] |  |  |
|  |  |  |  |  |  |  |  |  |  |  |  |  |  |  |  |  |  |  |  |
| 17. PHYAS_AGE9 | .53 | .71 | .15** | .12** | -.14** | .23** | .46** | .08 | .12** | .16** | -.21** | .28** | .58** | .06 | .26** | .27** | -.37** | .58** |  |
|  |  |  | [.06, .24] | [.04, .21] | [-.23, -.05] | [.14, .31] | [.38, .53] | [-.02, .17] | [.03, .21] | [.08, .25] | [-.30, -.12] | [.19, .36] | [.52, .64] | [-.04, .15] | [.18, .34] | [.19, .35] | [-.44, -.30] | [.52, .63] |  |
|  |  |  |  |  |  |  |  |  |  |  |  |  |  |  |  |  |  |  |  |
| 18. NEG_AGE9 | .11 | .30 | .03 | .16** | -.05 | .05 | .09 | .13** | .14** | .14** | .00 | .13** | .09 | .37** | .17** | .26** | -.08 | .25** | .20** |
|  |  |  | [-.06, .12] | [.08, .24] | [-.14, .04] | [-.04, .14] | [-.00, .18] | [.04, .22] | [.05, .23] | [.06, .23] | [-.09, .10] | [.03, .22] | [-.00, .19] | [.28, .45] | [.08, .25] | [.18, .34] | [-.16, .00] | [.17, .33] | [.12, .28] |
|  |  |  |  |  |  |  |  |  |  |  |  |  |  |  |  |  |  |  |  |

*Note.* *M* and *SD* are used to represent mean and standard deviation, respectively. Values in square brackets indicate the 95% confidence interval for each correlation. The confidence interval is a plausible range of population correlations that could have caused the sample correlation (Cumming, 2014). * indicates *p* < .05. ** indicates *p* < .01. IRR = child irritability, PSTR = parenting stress, NVIO = non-violent discipline, PSYAGG = psychological aggression, PHYAS = physical assault, NEG = neglect.

**Table S4**

*Means, standard deviations, and correlations with confidence intervals for Black participants at all timepoints*

| Variable | *M* | *SD* | 1 | 2 | 3 | 4 | 5 | 6 | 7 | 8 | 9 | 10 | 11 | 12 | 13 | 14 | 15 | 16 | 17 |
| --- | --- | --- | --- | --- | --- | --- | --- | --- | --- | --- | --- | --- | --- | --- | --- | --- | --- | --- | --- |
|  |  |  |  |  |  |  |  |  |  |  |  |  |  |  |  |  |  |  |  |
| 1. IRR_AGE3 | .68 | .55 |  |  |  |  |  |  |  |  |  |  |  |  |  |  |  |  |  |
|  |  |  |  |  |  |  |  |  |  |  |  |  |  |  |  |  |  |  |  |
| 2. PSTR_AGE3 | 2.26 | .66 | .20** |  |  |  |  |  |  |  |  |  |  |  |  |  |  |  |  |
|  |  |  | [.14, .26] |  |  |  |  |  |  |  |  |  |  |  |  |  |  |  |  |
|  |  |  |  |  |  |  |  |  |  |  |  |  |  |  |  |  |  |  |  |
| 3. NVIO_AGE3 | 1.84 | 1.33 | -.13** | -.07* |  |  |  |  |  |  |  |  |  |  |  |  |  |  |  |
|  |  |  | [-.19, -.07] | [-.13, -.01] |  |  |  |  |  |  |  |  |  |  |  |  |  |  |  |
|  |  |  |  |  |  |  |  |  |  |  |  |  |  |  |  |  |  |  |  |
| 4. PSYAGG_AGE3 | 1.75 | .97 | .27** | .15** | -.43** |  |  |  |  |  |  |  |  |  |  |  |  |  |  |
|  |  |  | [.21, .32] | [.09, .21] | [-.48, -.38] |  |  |  |  |  |  |  |  |  |  |  |  |  |  |
|  |  |  |  |  |  |  |  |  |  |  |  |  |  |  |  |  |  |  |  |
| 5. PHYAS_AGE3 | 1.48 | 1.06 | .22** | .14** | -.39** | .61** |  |  |  |  |  |  |  |  |  |  |  |  |  |
|  |  |  | [.16, .28] | [.08, .20] | [-.44, -.34] | [.56, .64] |  |  |  |  |  |  |  |  |  |  |  |  |  |
|  |  |  |  |  |  |  |  |  |  |  |  |  |  |  |  |  |  |  |  |
| 6. NEG_AGE3 | .07 | .31 | .12** | .11** | -.01 | .12** | .12** |  |  |  |  |  |  |  |  |  |  |  |  |
|  |  |  | [.05, .18] | [.05, .17] | [-.07, .06] | [.06, .18] | [.06, .18] |  |  |  |  |  |  |  |  |  |  |  |  |
|  |  |  |  |  |  |  |  |  |  |  |  |  |  |  |  |  |  |  |  |
| 7. IRR_AGE5 | .55 | .52 | .44** | .19** | .02 | .15** | .13** | .06 |  |  |  |  |  |  |  |  |  |  |  |
|  |  |  | [.39, .49] | [.13, .25] | [-.05, .08] | [.08, .21] | [.06, .19] | [-.00, .13] |  |  |  |  |  |  |  |  |  |  |  |
|  |  |  |  |  |  |  |  |  |  |  |  |  |  |  |  |  |  |  |  |
| 8. PSTR_AGE5 | 2.18 | .69 | .18** | .57** | -.08* | .12** | .12** | .06 | .28** |  |  |  |  |  |  |  |  |  |  |
|  |  |  | [.12, .24] | [.53, .61] | [-.14, -.01] | [.06, .19] | [.05, .18] | [-.00, .12] | [.23, .34] |  |  |  |  |  |  |  |  |  |  |
|  |  |  |  |  |  |  |  |  |  |  |  |  |  |  |  |  |  |  |  |
| 9. NVIO_AGE5 | 1.72 | 1.29 | -.12** | -.07* | .38** | -.24** | -.24** | -.05 | -.16** | -.15** |  |  |  |  |  |  |  |  |  |
|  |  |  | [-.18, -.05] | [-.13, -.00] | [.32, .43] | [-.30, -.17] | [-.30, -.17] | [-.11, .02] | [-.22, -.10] | [-.22, -.09] |  |  |  |  |  |  |  |  |  |
|  |  |  |  |  |  |  |  |  |  |  |  |  |  |  |  |  |  |  |  |
| 10. PSYAGG_AGE5 | 1.86 | 1.00 | .17** | .15** | -.23** | .48** | .32** | .09* | .22** | .19** | -.46** |  |  |  |  |  |  |  |  |
|  |  |  | [.11, .24] | [.08, .21] | [-.29, -.16] | [.43, .53] | [.26, .38] | [.02, .15] | [.16, .28] | [.12, .25] | [-.51, -.41] |  |  |  |  |  |  |  |  |
|  |  |  |  |  |  |  |  |  |  |  |  |  |  |  |  |  |  |  |  |
| 11. PHYAS_AGE5 | 1.32 | 1.00 | .16** | .12** | -.20** | .36** | .50** | .13** | .18** | .17** | -.41** | .60** |  |  |  |  |  |  |  |
|  |  |  | [.09, .22] | [.05, .18] | [-.27, -.14] | [.30, .42] | [.45, .55] | [.06, .19] | [.12, .24] | [.11, .23] | [-.46, -.36] | [.55, .63] |  |  |  |  |  |  |  |
|  |  |  |  |  |  |  |  |  |  |  |  |  |  |  |  |  |  |  |  |
| 12. NEG_AGE5 | .06 | .21 | .06 | .12** | .04 | .04 | .08* | .14** | .05 | .14** | .01 | .12** | .11** |  |  |  |  |  |  |
|  |  |  | [-.01, .13] | [.06, .19] | [-.03, .11] | [-.03, .11] | [.01, .14] | [.07, .20] | [-.02, .11] | [.08, .20] | [-.06, .07] | [.06, .19] | [.05, .18] |  |  |  |  |  |  |
|  |  |  |  |  |  |  |  |  |  |  |  |  |  |  |  |  |  |  |  |
| 13. IRR_AGE9 | .33 | .42 | .33** | .13** | -.06 | .11** | .08* | .01 | .40** | .22** | -.12** | .19** | .19** | .03 |  |  |  |  |  |
|  |  |  | [.27, .39] | [.07, .19] | [-.12, .01] | [.05, .18] | [.02, .15] | [-.06, .07] | [.34, .45] | [.16, .28] | [-.19, -.06] | [.12, .25] | [.12, .25] | [-.03, .10] |  |  |  |  |  |
|  |  |  |  |  |  |  |  |  |  |  |  |  |  |  |  |  |  |  |  |
| 14. PSTR_AGE9 | 2.08 | .70 | .09** | .40** | -.07* | .10** | .13** | .04 | .14** | .49** | -.08* | .12** | .17** | .08* | .19** |  |  |  |  |
|  |  |  | [.03, .15] | [.35, .45] | [-.13, -.01] | [.04, .17] | [.06, .19] | [-.02, .11] | [.08, .20] | [.44, .53] | [-.14, -.01] | [.06, .19] | [.10, .23] | [.01, .14] | [.13, .24] |  |  |  |  |
|  |  |  |  |  |  |  |  |  |  |  |  |  |  |  |  |  |  |  |  |
| 15. NVIO_AGE9 | 2.41 | 1.41 | -.10** | -.10** | .37** | -.25** | -.26** | -.03 | -.12** | -.08** | .42** | -.32** | -.31** | -.02 | -.24** | -.12** |  |  |  |
|  |  |  | [-.16, -.03] | [-.16, -.04] | [.31, .43] | [-.31, -.19] | [-.32, -.20] | [-.09, .04] | [-.18, -.06] | [-.14, -.02] | [.37, .48] | [-.37, -.25] | [-.37, -.25] | [-.09, .04] | [-.30, -.18] | [-.18, -.06] |  |  |  |
|  |  |  |  |  |  |  |  |  |  |  |  |  |  |  |  |  |  |  |  |
| 16. PSYAGG_AGE9 | 1.58 | 1.09 | .17** | .16** | -.20** | .39** | .30** | .01 | .21** | .17** | -.29** | .48** | .37** | .11** | .32** | .19** | -.58** |  |  |
|  |  |  | [.10, .23] | [.10, .22] | [-.26, -.13] | [.33, .44] | [.24, .36] | [-.05, .08] | [.15, .27] | [.11, .23] | [-.35, -.23] | [.42, .53] | [.31, .42] | [.05, .18] | [.26, .37] | [.13, .25] | [-.62, -.54] |  |  |
|  |  |  |  |  |  |  |  |  |  |  |  |  |  |  |  |  |  |  |  |
| 17. PHYAS_AGE9 | .90 | .96 | .17** | .13** | -.17** | .22** | .36** | .07* | .16** | .12** | -.25** | .31** | .45** | .06 | .30** | .16** | -.51** | .60** |  |
|  |  |  | [.11, .23] | [.07, .19] | [-.24, -.11] | [.16, .29] | [.30, .42] | [.01, .14] | [.10, .23] | [.06, .18] | [-.31, -.19] | [.25, .37] | [.40, .51] | [-.00, .13] | [.25, .36] | [.10, .21] | [-.55, -.46] | [.56, .64] |  |
|  |  |  |  |  |  |  |  |  |  |  |  |  |  |  |  |  |  |  |  |
| 18. NEG_AGE9 | .14 | .42 | .12** | .08** | -.04 | .10** | .09** | .10** | .06 | .10** | -.06 | .12** | .10** | .15** | .16** | .09** | -.09** | .23** | .20** |
|  |  |  | [.06, .19] | [.02, .15] | [-.11, .02] | [.03, .16] | [.02, .15] | [.03, .16] | [-.01, .12] | [.03, .16] | [-.13, .01] | [.05, .18] | [.03, .16] | [.08, .21] | [.10, .21] | [.03, .15] | [-.15, -.03] | [.17, .29] | [.14, .26] |
|  |  |  |  |  |  |  |  |  |  |  |  |  |  |  |  |  |  |  |  |

*Note.* *M* and *SD* are used to represent mean and standard deviation, respectively. Values in square brackets indicate the 95% confidence interval for each correlation. The confidence interval is a plausible range of population correlations that could have caused the sample correlation (Cumming, 2014). * indicates *p* < .05. ** indicates *p* < .01. IRR = child irritability, PSTR = parenting stress, NVIO = non-violent discipline, PSYAGG = psychological aggression, PHYAS = physical assault, NEG = neglect.

**Table S5**

*Means, standard deviations, and correlations with confidence intervals for Latine participants at all timepoints*

| Variable | *M* | *SD* | 1 | 2 | 3 | 4 | 5 | 6 | 7 | 8 | 9 | 10 | 11 | 12 | 13 | 14 | 15 | 16 | 17 |
| --- | --- | --- | --- | --- | --- | --- | --- | --- | --- | --- | --- | --- | --- | --- | --- | --- | --- | --- | --- |
|  |  |  |  |  |  |  |  |  |  |  |  |  |  |  |  |  |  |  |  |
| 1. IRR_AGE3 | .59 | .48 |  |  |  |  |  |  |  |  |  |  |  |  |  |  |  |  |  |
|  |  |  |  |  |  |  |  |  |  |  |  |  |  |  |  |  |  |  |  |
| 2. PSTR_AGE3 | 2.20 | .69 | .12** |  |  |  |  |  |  |  |  |  |  |  |  |  |  |  |  |
|  |  |  | [.04, .20] |  |  |  |  |  |  |  |  |  |  |  |  |  |  |  |  |
|  |  |  |  |  |  |  |  |  |  |  |  |  |  |  |  |  |  |  |  |
| 3. NVIO_AGE3 | 2.16 | 1.44 | -.25** | -.08 |  |  |  |  |  |  |  |  |  |  |  |  |  |  |  |
|  |  |  | [-.33, -.17] | [-.16, .00] |  |  |  |  |  |  |  |  |  |  |  |  |  |  |  |
|  |  |  |  |  |  |  |  |  |  |  |  |  |  |  |  |  |  |  |  |
| 4. PSYAGG_AGE3 | 1.37 | .92 | .31** | .22** | -.44** |  |  |  |  |  |  |  |  |  |  |  |  |  |  |
|  |  |  | [.23, .38] | [.14, .30] | [-.51, -.37] |  |  |  |  |  |  |  |  |  |  |  |  |  |  |
|  |  |  |  |  |  |  |  |  |  |  |  |  |  |  |  |  |  |  |  |
| 5. PHYAS_AGE3 | .90 | .85 | .27** | .12** | -.43** | .64** |  |  |  |  |  |  |  |  |  |  |  |  |  |
|  |  |  | [.19, .35] | [.04, .20] | [-.49, -.36] | [.59, .69] |  |  |  |  |  |  |  |  |  |  |  |  |  |
|  |  |  |  |  |  |  |  |  |  |  |  |  |  |  |  |  |  |  |  |
| 6. NEG_AGE3 | .08 | .36 | .04 | .08* | -.08 | .11* | .07 |  |  |  |  |  |  |  |  |  |  |  |  |
|  |  |  | [-.05, .12] | [.00, .17] | [-.16, .00] | [.02, .19] | [-.01, .16] |  |  |  |  |  |  |  |  |  |  |  |  |
|  |  |  |  |  |  |  |  |  |  |  |  |  |  |  |  |  |  |  |  |
| 7. IRR_AGE5 | .50 | .50 | .39** | .09 | -.09 | .12* | .14** | .01 |  |  |  |  |  |  |  |  |  |  |  |
|  |  |  | [.31, .47] | [-.00, .17] | [-.18, .00] | [.03, .21] | [.05, .23] | [-.08, .10] |  |  |  |  |  |  |  |  |  |  |  |
|  |  |  |  |  |  |  |  |  |  |  |  |  |  |  |  |  |  |  |  |
| 8. PSTR_AGE5 | 2.11 | .69 | .12** | .53** | -.03 | .16** | .09* | .09* | .25** |  |  |  |  |  |  |  |  |  |  |
|  |  |  | [.04, .21] | [.47, .59] | [-.12, .06] | [.07, .24] | [.00, .17] | [.01, .18] | [.17, .33] |  |  |  |  |  |  |  |  |  |  |
|  |  |  |  |  |  |  |  |  |  |  |  |  |  |  |  |  |  |  |  |
| 9. NVIO_AGE5 | 2.01 | 1.39 | -.25** | -.09 | .47** | -.27** | -.29** | -.03 | -.16** | -.09 |  |  |  |  |  |  |  |  |  |
|  |  |  | [-.33, -.15] | [-.18, .00] | [.40, .55] | [-.36, -.18] | [-.37, -.20] | [-.13, .06] | [-.25, -.07] | [-.17, .01] |  |  |  |  |  |  |  |  |  |
|  |  |  |  |  |  |  |  |  |  |  |  |  |  |  |  |  |  |  |  |
| 10. PSYAGG_AGE5 | 1.57 | 1.00 | .32** | .24** | -.32** | .51** | .45** | .11* | .18** | .19** | -.44** |  |  |  |  |  |  |  |  |
|  |  |  | [.23, .40] | [.15, .32] | [-.41, -.23] | [.43, .57] | [.37, .53] | [.02, .21] | [.09, .27] | [.10, .27] | [-.51, -.36] |  |  |  |  |  |  |  |  |
|  |  |  |  |  |  |  |  |  |  |  |  |  |  |  |  |  |  |  |  |
| 11. PHYAS_AGE5 | .89 | .91 | .23** | .21** | -.25** | .35** | .45** | .16** | .16** | .19** | -.40** | .62** |  |  |  |  |  |  |  |
|  |  |  | [.13, .32] | [.12, .29] | [-.34, -.16] | [.26, .43] | [.37, .52] | [.06, .25] | [.07, .25] | [.10, .28] | [-.48, -.32] | [.57, .68] |  |  |  |  |  |  |  |
|  |  |  |  |  |  |  |  |  |  |  |  |  |  |  |  |  |  |  |  |
| 12. NEG_AGE5 | .06 | .21 | .16** | .09 | -.03 | .15** | .10* | .11* | .14** | .12** | -.09* | .19** | .15** |  |  |  |  |  |  |
|  |  |  | [.07, .26] | [-.00, .18] | [-.12, .07] | [.05, .24] | [.01, .20] | [.02, .21] | [.05, .23] | [.03, .21] | [-.18, -.00] | [.10, .28] | [.06, .23] |  |  |  |  |  |  |
|  |  |  |  |  |  |  |  |  |  |  |  |  |  |  |  |  |  |  |  |
| 13. IRR_AGE9 | .27 | .40 | .32** | .04 | -.11* | .10* | .15** | .05 | .41** | .08 | -.20** | .23** | .21** | .17** |  |  |  |  |  |
|  |  |  | [.24, .40] | [-.05, .12] | [-.20, -.02] | [.01, .19] | [.07, .24] | [-.04, .14] | [.32, .48] | [-.01, .16] | [-.29, -.11] | [.13, .32] | [.12, .30] | [.08, .26] |  |  |  |  |  |
|  |  |  |  |  |  |  |  |  |  |  |  |  |  |  |  |  |  |  |  |
| 14. PSTR_AGE9 | 1.90 | .67 | .17** | .38** | -.04 | .17** | .08 | .02 | .18** | .43** | -.16** | .24** | .20** | .07 | .18** |  |  |  |  |
|  |  |  | [.09, .26] | [.30, .45] | [-.13, .05] | [.08, .26] | [-.01, .17] | [-.07, .11] | [.09, .27] | [.36, .50] | [-.25, -.06] | [.15, .33] | [.11, .29] | [-.02, .17] | [.10, .26] |  |  |  |  |
|  |  |  |  |  |  |  |  |  |  |  |  |  |  |  |  |  |  |  |  |
| 15. NVIO_AGE9 | 2.96 | 1.57 | -.20** | -.03 | .49** | -.27** | -.34** | -.03 | -.14** | -.02 | .52** | -.36** | -.31** | -.02 | -.29** | -.17** |  |  |  |
|  |  |  | [-.28, -.11] | [-.12, .05] | [.42, .56] | [-.35, -.19] | [-.42, -.25] | [-.12, .06] | [-.23, -.05] | [-.10, .07] | [.45, .59] | [-.44, -.27] | [-.39, -.22] | [-.12, .08] | [-.37, -.21] | [-.25, -.09] |  |  |  |
|  |  |  |  |  |  |  |  |  |  |  |  |  |  |  |  |  |  |  |  |
| 16. PSYAGG_AGE9 | 1.21 | 1.00 | .15** | .13** | -.31** | .36** | .35** | .01 | .12* | .10* | -.30** | .46** | .37** | .07 | .32** | .18** | -.62** |  |  |
|  |  |  | [.06, .24] | [.04, .21] | [-.39, -.22] | [.28, .44] | [.26, .42] | [-.08, .11] | [.03, .21] | [.02, .19] | [-.39, -.21] | [.38, .53] | [.29, .45] | [-.02, .17] | [.25, .40] | [.10, .26] | [-.67, -.56] |  |  |
|  |  |  |  |  |  |  |  |  |  |  |  |  |  |  |  |  |  |  |  |
| 17. PHYAS_AGE9 | .59 | .74 | .13** | .13** | -.20** | .23** | .35** | .02 | .14** | .11* | -.18** | .30** | .37** | .03 | .26** | .19** | -.47** | .63** |  |
|  |  |  | [.04, .22] | [.04, .21] | [-.29, -.11] | [.14, .31] | [.26, .42] | [-.07, .11] | [.04, .23] | [.02, .19] | [-.27, -.08] | [.21, .39] | [.29, .45] | [-.06, .13] | [.18, .33] | [.11, .27] | [-.53, -.40] | [.58, .68] |  |
|  |  |  |  |  |  |  |  |  |  |  |  |  |  |  |  |  |  |  |  |
| 18. NEG_AGE9 | .15 | .37 | .07 | .10* | -.04 | .10* | .09* | .09* | .01 | .13** | -.04 | .12* | .10* | .17** | .10* | .15** | -.12** | .25** | .23** |
|  |  |  | [-.02, .16] | [.01, .18] | [-.13, .05] | [.01, .19] | [.00, .18] | [.00, .18] | [-.09, .10] | [.04, .21] | [-.14, .05] | [.02, .21] | [.01, .20] | [.07, .26] | [.02, .19] | [.07, .24] | [-.20, -.04] | [.17, .33] | [.15, .31] |
|  |  |  |  |  |  |  |  |  |  |  |  |  |  |  |  |  |  |  |  |

*Note.* *M* and *SD* are used to represent mean and standard deviation, respectively. Values in square brackets indicate the 95% confidence interval for each correlation. The confidence interval is a plausible range of population correlations that could have caused the sample correlation (Cumming, 2014). * indicates *p* < .05. ** indicates *p* < .01. IRR = child irritability, PSTR = parenting stress, NVIO = non-violent discipline, PSYAGG = psychological aggression, PHYAS = physical assault, NEG = neglect.

**Table S6.** Network comparison tests comparing differences in edge weights of one racial-ethnic group at two timepoints, for all cross-sectional networks.

|  | Age 3 – 5 White | Age 5 - 9 White | Age 3 – 9 White | Age 3 – 5 Black | Age 5 – 9 Black | Age 3 – 9 Black | Age 3 – 5 Latine | Age 5 - 9 Latine | Age 3 – 9 Latine |
| --- | --- | --- | --- | --- | --- | --- | --- | --- | --- |
| irr - pstr | .289*** | - | .180** | .141** | .145*** | - | .233* | - | .198* |
| irr - nVio | - | - | - | - | .138*** | - | - | .230*** | .230* |
| pstr - nVio | - | - | - | - | .087* | - | - | - | - |
| irr - psyAgg | .211* | - | - | - | - | - | - | .171* | - |
| pstr - psyAgg | - | - | - | - | - | - | .137** | - | - |
| nVio - psyAgg | .017* | .002* | - | - | .141** | .122*** | - | - | .146** |
| irr - phyAs | - | - | - | - | .171** | - | - | - | - |
| pstr - phyAs | - | - | - | - | - | - | - | .115* | - |
| nVio - phyAs | - | - | - | .087*** | - | .087** | - | - | - |
| psyAgg - phyAs | - | .076* | - | - | .076* | - | - | .108** | - |
| irr - neg | .244*** | .227** | - | .230*** | .169* | - | .221*** | - | .221** |
| pstr - neg | - | .122* | - | - | .151** | - | - | .210* | .210** |
| nVio - neg | .197*** | .203** | - | - | .241*** | .177*** | - | .135* | - |
| psyAgg – neg | .125* | - | - | .086 * | - | - | - | - | - |
| phyAs - neg | - | - | - | - | .109* | - | - | - | - |
| irr - cAtt | - | .112** | .088* | .112** | - | - | - | - | - |
| pstr - cAtt | - | - | - | - | - | - | - | - | - |
| nVio - cAtt | - | - | - | - | - | - | - | - | - |
| psyAgg - cAtt | - | - | - | - | - | - | - | - | - |
| phyAs - cAtt | - | - | - | - | .091* | .091* | - | - | - |
| neg - cAtt | - | - | - | - | - | - | - | - | - |
| irr - cPra | - | - | - | - | - | - | - | - | - |
| pstr - cPra | - | - | - | - | - | - | - | - | - |
| nVio - cPra | - | - | - | - | - | - | - | - | - |
| psyAgg - cPra | - | - | - | - | - | - | - | - | - |
| phyAs - cPra | - | - | - | - | - | - | .101** | - | .101** |
| neg - cPra | - | - | - | - | - | - | - | - | - |
| cAtt - cPra | - | - | - | - | - | - | - | - | - |

**Note**. Values represent absolute edge differences [AED] between timepoints. irr = child irritability; pstr = parenting stress; nVio = non-violent discipline; psyAgg = psychological aggression; phyAs = physical assault; neg = neglect; cAtt = mother cultural attachment; cPra = mother cultural practice. ^ = *p*<.1, * = *p*<.05, ** = *p*<.01, *** = *p*<.001, absent values = no significant differences

**Table S7. Specific edge differences between the longitudinal networks.** B = Black participants; W = White participants; L = Latine participants; red highlighted rows represent significant differences

| Specific path differences | | | | | | |
| --- | --- | --- | --- | --- | --- | --- |
| Path | B vs W | | B v L | | W vs L | |
|  | Δ edge strength | *P* | Δ edge strength | *P* | Δ edge strength | *P* |
| pstr -> pstr | -.075 | .508 | .139 | .559 | .214 | .258 |
| pstr -> irr | .146 | .226 | .153 | .215 | .007 | .962 |
| pstr -> neg | .203 | .573 | .136 | .771 | -.067 | .774 |
| pstr -> nVio | -.107 | .864 | .187 | .230 | .294 | .173 |
| pstr -> phyAs | .306 | .141 | .265 | .194 | -.041 | .803 |
| pstr -> psyAgg | .146 | .690 | .181 | .546 | .035 | .833 |
| irr -> pstr | -.004 | .868 | -.108 | .352 | -.104 | .433 |
| irr -> irr | .070 | .928 | -.133 | .355 | -.203 | .362 |
| irr -> neg | -.535 | .059 | -.223 | .388 | .312 | .237 |
| irr -> nVio | .233 | .344 | -.072 | .886 | -.305 | .214 |
| irr -> phyAs | -.145 | .407 | -.230 | .225 | -.086 | .652 |
| irr -> psyAgg | -.409 | .097 | -.205 | .350 | .204 | .374 |
| neg -> pstr | .046 | .548 | -.195 | .229 | -.242 | .121 |
| neg -> irr | .011 | 1.000 | .043 | .858 | .032 | .832 |
| neg -> neg | .013 | .717 | -.249 | .550 | -.262 | .347 |
| neg -> nVio | .071 | 1.000 | -.140 | .353 | -.211 | .433 |
| neg -> phyAs | -.463 | .104 | -.385 | .182 | .077 | .730 |
| neg -> psyAgg | -.222 | .636 | -.210 | .677 | .012 | .943 |
| nVio -> pstr | .080 | .545 | .139 | .227 | .058 | .592 |
| nVio -> irr | -.064 | .582 | -.075 | .512 | -.011 | .916 |
| nVio -> neg | -.028 | .702 | .040 | .986 | .068 | .709 |
| nVio -> nVio | -.360 | .074 | -.416 | .039 | -.056 | .728 |
| nVio -> phyAs | .435 | .043 | .359 | .074 | -.076 | .598 |
| nVio -> psyAgg | .346 | .086 | .476 | .020 | .130 | .403 |
| phyAs -> pstr | -.006 | .923 | .037 | .758 | .042 | .704 |
| phyAs -> irr | -.128 | .247 | .015 | 1.000 | .142 | .300 |
| phyAs -> neg | .070 | .638 | -.012 | 1.000 | -.082 | .745 |
| phyAs -> nVio | -.077 | .714 | -.086 | .694 | -.010 | .986 |
| phyAs -> phyAs | -.047 | .702 | .219 | .430 | .265 | .249 |
| phyAs -> psyAgg | .082 | .779 | .066 | .851 | -.015 | .939 |
| psyAgg -> pstr | -.162 | .103 | .121 | .372 | .283 | .039 |
| psyAgg -> irr | -.052 | .716 | -.031 | .858 | .021 | .854 |
| psyAgg -> neg | .034 | 1.000 | .155 | .762 | .122 | .612 |
| psyAgg -> nVio | -.019 | 1.000 | .096 | .370 | .115 | .591 |
| psyAgg -> phyAs | .353 | .125 | .292 | .204 | -.061 | .738 |
| psyAgg -> psyAgg | .106 | .948 | .182 | .615 | .076 | .698 |

**Table S8. Bootstrap edge inclusion rate in 1000 iterations, for Black participant variables.** Edges with a value greater than .5 were included in the longitudinal model.

|  | Irr | Pstr | nVio | PsyAgg | PhyAs | Neg |
| --- | --- | --- | --- | --- | --- | --- |
| Irr | .835 | .766 | .391 | .595 | .619 | .453 |
| Pstr | .902 | .964 | .558 | .791 | .653 | .727 |
| nVio | .867 | .899 | .218 | .827 | .914 | .543 |
| PsyAgg | .606 | .577 | .265 | .855 | .697 | .743 |
| PhyAs | .755 | .626 | .324 | .769 | .854 | .372 |
| Neg | .314 | .211 | .687 | .184 | .170 | .390 |

**Table S9. Bootstrap edge inclusion rate in 1000 iterations, for White participant variables.** Edges with a value greater than .5 were included in the longitudinal model.

|  | Irr | Pstr | nVio | PsyAgg | PhyAs | Neg |
| --- | --- | --- | --- | --- | --- | --- |
| Irr | .830 | .366 | .141 | .917 | .414 | .798 |
| Pstr | .211 | .913 | .443 | .857 | .267 | .532 |
| nVio | .755 | .777 | .741 | .799 | .301 | .886 |
| PsyAgg | .619 | .896 | .103 | .892 | .249 | .722 |
| PhyAs | .929 | .755 | .367 | .853 | .962 | .357 |
| Neg | .608 | .258 | .870 | .159 | .812 | .285 |

**Table S10. Bootstrap edge inclusion rate in 1000 iterations, for Latine participant variables.** Edges with a value greater than .5 were included in the longitudinal model.

|  | Irr | Pstr | nVio | PsyAgg | PhyAs | Neg |
| --- | --- | --- | --- | --- | --- | --- |
| Irr | .862 | .819 | .463 | .668 | .706 | .488 |
| Pstr | .312 | .825 | .203 | .690 | .560 | .684 |
| nVio | .615 | .642 | .437 | .631 | .676 | .626 |
| PsyAgg | .589 | .472 | .129 | .857 | .540 | .770 |
| PhyAs | .684 | .545 | .439 | .851 | .800 | .300 |
| Neg | .447 | .596 | .860 | .178 | .536 | .400 |
